# Estrogen regulates divergent transcriptional and epigenetic cell states in breast cancer

**DOI:** 10.1093/nar/gkac908

**Published:** 2022-11-01

**Authors:** Aysegul Ors, Alex Daniel Chitsazan, Aaron Reid Doe, Ryan M Mulqueen, Cigdem Ak, Yahong Wen, Syber Haverlack, Mithila Handu, Spandana Naldiga, Joshua C Saldivar, Hisham Mohammed

**Affiliations:** Cancer Early Detection Advanced Research Center, Knight Cancer Institute, Oregon Health & Science University, Portland, Oregon 97201, USA; Cancer Early Detection Advanced Research Center, Knight Cancer Institute, Oregon Health & Science University, Portland, Oregon 97201, USA; Cancer Early Detection Advanced Research Center, Knight Cancer Institute, Oregon Health & Science University, Portland, Oregon 97201, USA; Cancer Early Detection Advanced Research Center, Knight Cancer Institute, Oregon Health & Science University, Portland, Oregon 97201, USA; Cancer Early Detection Advanced Research Center, Knight Cancer Institute, Oregon Health & Science University, Portland, Oregon 97201, USA; Cancer Early Detection Advanced Research Center, Knight Cancer Institute, Oregon Health & Science University, Portland, Oregon 97201, USA; Cancer Early Detection Advanced Research Center, Knight Cancer Institute, Oregon Health & Science University, Portland, Oregon 97201, USA; Cancer Early Detection Advanced Research Center, Knight Cancer Institute, Oregon Health & Science University, Portland, Oregon 97201, USA; Cancer Early Detection Advanced Research Center, Knight Cancer Institute, Oregon Health & Science University, Portland, Oregon 97201, USA; Cancer Early Detection Advanced Research Center, Knight Cancer Institute, Oregon Health & Science University, Portland, Oregon 97201, USA; Division of Oncological Sciences, Knight Cancer Institute, Oregon Health & Science University, Portland, Oregon 97201, USA; Cancer Early Detection Advanced Research Center, Knight Cancer Institute, Oregon Health & Science University, Portland, Oregon 97201, USA; Department of Molecular and Medical Genetics, Oregon Health & Science University, Portland, Oregon 97201, USA

## Abstract

Breast cancers are known to be driven by the transcription factor estrogen receptor and its ligand estrogen. While the receptor's cis-binding elements are known to vary between tumors, heterogeneity of hormone signaling at a single-cell level is unknown. In this study, we systematically tracked estrogen response across time at a single-cell level in multiple cell line and organoid models. To accurately model these changes, we developed a computational tool (TITAN) that quantifies signaling gradients in single-cell datasets. Using this approach, we found that gene expression response to estrogen is non-uniform, with distinct cell groups expressing divergent transcriptional networks. Pathway analysis suggested the two most distinct signatures are driven separately by ER and FOXM1. We observed that FOXM1 was indeed activated by phosphorylation upon estrogen stimulation and silencing of FOXM1 attenuated the relevant gene signature. Analysis of scRNA-seq data from patient samples confirmed the existence of these divergent cell groups, with the FOXM1 signature predominantly found in ER negative cells. Further, multi-omic single-cell experiments indicated that the different cell groups have distinct chromatin accessibility states. Our results provide a comprehensive insight into ER biology at the single-cell level and potential therapeutic strategies to mitigate resistance to therapy.

## INTRODUCTION

Estrogen receptor alpha (ER) is essential for the development and homeostasis of the mammary gland and is known to drive up to 70% of all breast cancers. ER is a transcription factor, that upon activation by its ligand, the hormone estrogen, binds to chromatin, modulating gene expression. ER activity across time is not uniform, with ER binding and cofactor recruitment shown to cycle at regular time intervals (1,2). Additionally, other hormone receptors such as the progesterone, androgen and glucocorticoid receptors have been shown by us and others to influence ER’s chromatin binding patterns (3–8). It is unknown whether response to hormone is uniform across all cells in a system and whether heterogeneity in response rates have any biological consequences. Time resolved single-cell studies of in-vitro model systems can hence better fundamental understanding of hormone signaling dynamics. Single-cell omics in recent years demonstrated the highly heterogeneous nature of tumors at both the transcriptional and genetic levels (9–13), significantly contributing to our understanding of tumor evolution and drug resistance (14). The ‘cell state’ model proposed in tumors suggests the existence of fluctuating transcriptional states within the system, allowing potential escape mechanisms under therapeutic pressures. Distinct transcriptional and epigenetic cell states have been shown to exist across breast cancer development in mouse tumor models using single-cell RNA-seq and bulk ATAC-sequencing approaches (15,16). In human breast tumors, Wu *et al.* recently reported that the PAM50 subtype classification of breast tumors at a single-cell level identified that cells within a single tumor demonstrate signatures across multiple breast cancer subtypes (13). They identified multiple transcriptional states in their studies and report marker gene characteristics of each state. From a data analysis perspective, while clustering has proved useful in defining distinct cell groups, such as the differentiated lineages in blood (17), identifying less discrete forms of heterogeneity, such as cell states or signaling gradients within a lineage have been challenging. Further, in developmental processes where transcriptional differences are often a unidirectional continuum, methods such as *monocle* and others (18–20) effectively allow recreation of these trajectories. However, these tools are inadequate to interrogate systems exhibiting high levels of plasticity such as embryonic stem cells or signaling networks in cancer. In these systems, transcriptional heterogeneity is less rigid and often occurs as multi-directional or cyclical gradients (2,21). In addition, some *a priori* knowledge is required about the system to give direction to trajectories limiting the ability to quantify *de novo* pathways and networks. This highlights the need for alternative computational approaches that allow the study of such latent transcriptional networks.

In this study, we finely map the heterogeneity of response to estrogen across time using single-cell RNA sequencing (scRNA-seq) and single-cell multiome (joint ATAC-RNA profiling) in multiple ER positive (ER+) breast cancer cell lines and patient-derived explant organoid (PdXO) models with the aim of identifying regulatory mechanisms driving heterogeneous hormone response rates. To analyze our findings, we developed a computational tool: TITAN (Topic Inference of Transcriptionally Associated Networks). TITAN is an unsupervised Bayesian topic modeling based approach utilizing latent Dirichlet allocation (LDA). TITAN distinguishes itself in its ability to assay distinct cell states that can be visualized against traditional clusters and UMAP based projections to infer signaling or other transcriptional gradients in a system. Additionally, TITAN uses natural language processing-based dimensionality reduction to aggregate signals into transcriptional networks, improving interpretability. Using TITAN, we find distinct estrogen responsive signatures, occurring in non-overlapping cell populations, also demonstrating distinct chromatin accessibility characteristics.

## MATERIALS AND METHODS

### Cell line maintenance

MCF-7, T-47D and ZR-75–1 cell lines were obtained from ATCC. MCF-7 cells were maintained in high glucose DMEM with L-glutamine and pyruvate (Gibco, 11995073) supplemented with 10% FBS (Gibco, A5209402) and 1% Penicillin-streptomycin (Gibco, 10378016). T-47D and ZR-75–1 cells were maintained in RPMI 1640 (Gibco, 11875093) supplemented with 10% FBS and 1% penicillin-streptomycin. Cells were grown at 37°C with 5% CO_2_. Monoclonal cell populations were selected from MCF-7 cells in culture using a limiting dilution approach. Distinct genomic differences were observed between the two clones used in this study by inferCNV analysis (Data not shown, https://github.com/broadinstitute/inferCNV). HCI003 and HCI011 PdXOs were obtained from the Welm lab and maintained in culture as described here (22).

### Hormone treatment for scRNA-seq and multiome experiments

Three days prior to hormone stimulation, cells or PdXO were transferred into their respective phenol-red free media supplemented with 5% charcoal-stripped FBS (Thermo Fisher Scientific, A3382101) and 1% Penicillin–streptomycin (Thermo Fisher Scientific, 15140122) and PdXO specific supplements (22). Culture media was refreshed 1 day prior to treatments. β-Estradiol (E2, Sigma-Aldrich, E2758), progesterone (PG, Sigma-Aldrich, P8783) or both hormones in combination were added at a final concentration of 100 nmol/l each for 3, 6, 24, 48 or 72 hours (h) as noted on figures. After treatment, cells were detached using phenol-red free TrypLE (Gibco, 12604013) and collected in 1X DPBS (Gibco, 14190250). Single cell suspensions from matrigel embedded PdXOs were obtained following dispase and TrypLE dissociation as described (22).

After one wash in 1× DPBS, cells were resuspended in 2% BSA–DPBS at a concentration of 10 000 cells/ml. Following incubation with TruStain FcX blocking solution (BioLegend, 422301), a Totalseq-A Hashtag antibody was added per treatment group. Cells were washed 3 times with 1 ml 2% BSA–DPBS and resuspended at a final concentration into a concentration of ∼10 000 cells/ml. Equal volumes of each treatment group were pooled and assessed for cell concentration and viability. Single-cell cDNA and hashtag barcode (HTO) libraries were generated using Chromium Single Cell 3′ version 3 and 3.1 reagents (10× genomics and Biolegend) per manufacturer's protocols. For multiome single cell data, cell lines were treated with 20 nmol/l β-estradiol and single-cell suspension was prepared as described previously. The Single Cell Multiome ATAC+ Gene Expression kit (10x Genomics) protocol was followed per manufacturer's guidelines to prepare nuclei, generate droplets and prepare single cell libraries from 16,000 nuclei per lane. Paired-end sequencing of libraries was performed per manufacturer's protocol on a Novaseq 6000 (Illumina) sequencer with HTO libraries constituting 5% of the sample.

### ScRNA-seq data alignment and quality control (QC)

*CellRanger* (v3.0.2) was used to align the raw reads to the human transcriptome (hg38) and create the gene by cell expression matrix using *CellRanger*’s ‘count’ function. The gene expression matrix was imported into R and the R toolkit *Seurat* (v4.0.3) was used for QC, dimensional reduction and cluster assignments (23,24). Cells with <500 detected genes were removed. The effects of cell cycle were regressed out using Seurat's pipeline. Cells were given a G2M-phase and S-phase score using Seurat's *CellCycleScoring* function. These two scores were then fed into *Seurat’*s Scale Data function as variables to regress out. Principal component analysis (PCA) was performed on the scaled gene expression data with the 2000 most variable genes used as input. Centred log ratio (CLR) normalization was performed on the HTO expression matrix and then the matrix was scaled (see detailed definition in the following section). Using *Seurat's HTODemux* function, samples were demultiplexed, thus connecting them to their treatment time point of origin. Once the cells have been identified, the gene expression matrices of cells of common origin (e.g. all of the MCF-7 cells that underwent an estrogen treatment) were integrated together using Seurat*’*s standard integration algorithm to remove any unwanted batch effect variation (25). Cell numbers that passed data quality control used in analysis were 14,788 cells for MCF-7 E2, 10,424 cells for T-47D E2, 9,433 cells for ZR-75-1 E2, 3,750 cells for TAMR, 1,615 cells for HCI003, 13,470 cells for HCI011, 15,315 cells for MCF-7 E2/PG, 10,668 cells for T-47D E2/PG and 9,458 cells for ZR-75–1 E2/PG data.

After integration, the cells were clustered in a two-step process. First, the cells were embedded in a k-nearest neighbor (KNN) graph and then, the Louvain algorithm was applied. The clustering was visualized using UMAP (Uniform Manifold Approximation and Projection). The marker genes that define the clusters were then found via *Seurat’*s *FindMarkers* function using the ‘wilcox’ method.

### Topic inference of transcriptionally associated networks (TITAN)

LDA was originally developed for text analysis. The model assumes that each document is a mixture of topics (i.e. a probability distribution with a Dirichlet prior) and each word is characterized by the document's topics. In the context of single-cell analysis, documents are considered as cells and genes are considered as words with gene expression levels becoming word frequencies. The resulting LDA for single-cell data is composed of a set of topic-to-cell and topic-to-gene distributions. Per-cell topic heatmaps can then be used as a low-dimensional embedding to investigate cell similarity and infer hierarchical relationships. Further analysis of topics can provide useful biological insights on the sets of genes driving the different stages of the process studied. *TITAN* is based on a topic modeling algorithm that involves running LDA with a collapsed Gibbs sampler for scRNA-seq, similar to the approach used by *cisTopic* for scATAC-seq (26).

LDA processes documents as a ‘bag of words’ model which means the frequency of the words in a document is considered independently of the order of the words. scATAC-seq data used in *cisTopic* is binary and counts the occurrence of the features however, scRNA-seq data is a count matrix. For this reason, the input data was transformed in such a way that highly expressed features won’t be over-represented in the topics. We used CLR transformation across features, (i) to have positive values which is a requirement for model inputs and (ii) to obtain scale-invariant values (i.e. the ratio of features is expected to remain the same regardless of total number of reads). Scale-invariant values help nullify the possible bias of highly variable read counts between individual cells.

*TITAN* has three main steps: (i) CLR normalization of the transcriptome matrix as input for LDA; (ii) LDA and (iii) model selection.

- CLR normalization: Centered log-ratio transformation is applied to each cell with given $G$ genes. We added a pseudo count to each component before the log transformation to ensure non-negativity. CLR-transformation is based on the ratio between the instant and its geometric mean $g( x ) = {( {{x_1} \ldots {x_G}} )^{\frac{1}{G}}}$ as follows:
  $$\begin{equation*}CLR\left( x \right) = \left[ {\ln \left( {\frac{{{x_i}}}{{g\left( x \right)}}} \right), \ldots ,\ln \left( {\frac{{{x_G}}}{{g\left( x \right)}}} \right)} \right].\end{equation*}$$
  Following CLR transformation into pseudo counts which converts abundance to ratios, we multiplied these by a scale factor (10 by default) then rounded the result to the nearest integer to input to LDA.
- LDA: RNA counts table for all given genes for a cell is regarded as an independent document. Genes in these documents are treated as words, which collectively constitute the vocabulary. A cell-gene (i.e. document-word) matrix is constructed from the RNA counts for given genes and used as input by the LDA algorithm.
  LDA derives, from the original high-dimensional and sparse data, (1) the probability distributions over the topics for each cell in the dataset ($\theta$) and (2) the probability distributions over the genes for each topic ($\phi$). These distributions indicate, respectively, how important a gene is for a cell ($\theta$), and how important genes are for the topic ($\phi$). Here, we used a collapsed Gibbs sampler in which we assign each gene in each cell to a certain topic by randomly sampling from a distribution where the probability of a gene being assigned to a topic is proportional to the contribution of that gene to the topic and the contribution of that topic to the cell.
  Given *N* cells, *G* expressed genes and a choice of *K* topics, the model is therefore made up of two sets of Dirichlet distributions:
  $$\begin{equation*}{\phi _k} \sim Dirichle{t_G}\left( \beta \right), \ \ k = 1 \ldots K\end{equation*}$$
  $$\begin{equation*}{\theta _d} \sim Dirichle{t_K}\left( \alpha \right), \ \ d = 1 \ldots N\end{equation*}$$
  where *α* and *β* are vectors of length *K* and *G* representing the priors of per-cell topics and per-topic genes, respectively. The use of smaller values of *α* and *β* makes it possible to control the sparsity of the model (i.e. the number of topics per cell and number of genes per topic). Then LDA models every cell using the following generative process:
  1. For a given cell $d$, the topic distribution, ${\theta _d} \sim Dirichle{t_K}( \alpha )$ is drawn.
  2. For the ${i^{th}}$ gene in the cell,
    - A topic assignment ${z_i} \sim {\theta _d}$ is drawn,
    - and a gene ${w_i} \sim {\phi _{{z_i}}}$ is drawn and observed.
- Model Selection: We used a heuristic approach to determine the appropriate number of topics required for each data. Multiple iterations of the LDA model are run with increasing numbers of topics. These are then used to generate a perplexity score for each model using the approach shown by Zhao *et al.* (27). Perplexity is a commonly used measurement of how well a probability distribution or probability model predicts a dataset. Lower perplexity indicates a better probabilistic model. For all given cells in the input data, perplexity is
  $$\begin{equation*}perplexity\left( {data} \right) = {e^{\frac{{\mathop \sum \nolimits_{d = 1}^N logp\,\left( {{w_d}} \right)}}{{\mathop \sum \nolimits_{d = 1}^N {G_d}}}}}\end{equation*}$$
  where ${G_d}$ is the number of genes in cell $d$.
  Plotting the rate of perplexity change (RPC) against the number of topics results in an elbow plot that defines an ‘elbow point’, the topic number at which the model reaches a low plateau. This point determines the optimal number of topics needed based on the data itself.

### Experimental model settings

To limit the feature-space of the genes and minimize noise, the features were limited to the most variable 5,000 genes using *Seurat*’s *FindVariableFeatures* function with ‘vst’ method.

The model was tested for ten different numbers of topics, from 10 to 100, in increments of 10, to create an elbow plot that plots the RPC as a function of numbers of topics as described in the previous section. The elbow plots for all the data analyzed in this paper can be found in Supplementary Figures S1A and S8A.

LDA has two hyperparameters, $\alpha$ and $\beta$. The first, $\alpha$, is the document-topic density, and affects how many topics will make up a single document. The second $\beta$ is the topic-word density and determines how many words will make up a single topic. After running through multiple iterations of the two hyperparameters with $\alpha$ ranging from 0.5 to 100 and $\beta$ ranging from .01 to 1, we determined that an $\alpha$ of 50 and a $\beta$ of 0.1 worked best in our data. Other studies have recommended an $\alpha$equal to 50 divided by the number of topics chosen ($T$) (26,28). Both hyperparameters can be changed.

### TF enrichment analysis

To find enrichment of TF (transcription factor) binding for each topic in our cell line data, the human factor ChIP-seq database from CistromeDB was used (29–31). Studies on MCF-7, T-47D and ZR-75-1 cell lines using total of 1160 studies, were compiled from the entire human factor database of ChIP-seq results.

Then, the top 2,000 peaks of TF enrichment in each study were compared to the top 50 genes in each topic using a hypergeometric test to test for significance of overlap using the R function *phyper* (see code in linked GitHub). Top factor was then associated with each topic by ascending order of significance.

### Topic transfer to new datasets

To transfer topics from dataset on to another, we first select the top 50 genes in each topic then calculate the gene signature score of these genes using *Seurat*’s *AddModuleScore* function which uses the method proposed originally by (32).

### Tool comparisons

TITAN was compared against three gene network analysis approaches, PCA-based UMAP/Louvain clustering and differentially expressed genes generated by Seurat, *SCENIC* (33) and *CountClust* (34). In order to achieve an accurate comparison, we identified estrogen signaling relevant outputs for each method compared. For *TITAN*, topics enriched with genes regulated by ERα (ESR1 topic) were identified by running the top 50 genes in each topic in Enrichr (35–37) in each cell line. These were topic 8 for MCF-7 E2, topic 20 for T-47D E2, and topic 8 for ZR-75–1 E2 datasets.

*Seurat*’s PCA-based UMAP visualization and cluster definition of scRNA-seq data is the most commonly used method to define transcriptional differences between groups. In each data set, the cluster demonstrating the highest increase in cell distribution in response to estrogen treatment was identified and then the average expression of the top 50 genes at the latest time point (72h) were computed for each cell line. These clusters were cluster 1 in MCF-7 E2, cluster 0 in T-47D E2 and cluster 1 in ZR-75–1 E2 datasets.

The second tool used for comparison is *SCENIC* which is designed to reconstruct gene regulatory networks (GRNs) in scRNA-seq data. Using a dataset, a motif ranking database and a list of known transcription factors as inputs, *SCENIC* outputs a list of regulons ranked by significance of enrichment. Regulons are described as co-expression modules with significant motif enrichment of the specific upstream regulator. Topics in TITAN can be compared to regulons. For the SCENIC based analysis, the full pipeline of *pySCENIC* (v0.10.3) was run. *GRNBoost2*, from the Python package *arboreto* (v0.1.5), was used to infer the co-expression network (33,38). The required transcription factor ranking database was downloaded from cisTargetDBs (hg38) (https://resources.aertslab.org/cistarget/). The required motif annotation table was downloaded from pySCENIC resources (https://resources.aertslab.org/cistarget/motif2tf/). Although the ESR1 regulon was not found to be significantly enriched as a result of the SCENIC analysis, the ESR1 regulon was added to the *z*-score comparison.

The third method we compared TITAN against, *CountClust* is an LDA based approach for scRNA-seq analysis. The *CountClust* clusters with the highest Spearman rank correlation across each dataset to the ESR1 topic defined by TITAN were selected and confirmed to be enriched with ESR1 target genes based on the CistromeDB database (Supplementary Figure S3B and C). These clusters were cluster 14 in MCF-7 E2, 5 in T-47D E2 and 12 in ZR-75-1 E2 datasets.

Lastly, to quantify response to estrogen across time for each method, we plotted a delta control Z-score which shows the distribution of cell scores as compared to a median baseline which is the median *Z*-score for the control timepoint.

### FOXM1 knock-down and qPCR

SMARTpools of ON-TARGETplus siRNAs were used to knockdown FOXM1 (Horizon discovery, L-009762–00-0005) and negative control siRNA (Qiagen, 1027310) was used as the control non-targeting siRNA. Cells were transferred to serum reduced and hormone deprived medium and treated with 20 nmol/l of ß-estradiol for 6 and 24 h as described above. Lipofection of siRNAs using Lipofectamine 3000 (Thermo Fisher Scientific, L3000001) was carried out per manufacturer's guidelines, 24 h before estrogen treatment. Samples were collected, homogenized and RNA was isolated using the Qiashredder (Qiagen, 79656) and RNA purification kits per manufacturer's guidelines (Qiagen, 74104). Quantitect reverse transcriptase kit (Qiagen, 205311) was used to generate cDNA per manufacturer's guidelines. For the quantitative PCR, Powerup Sybergreen Mastermix (Thermo Fisher Scientific, A25742) was used per manufacturer's guidelines for Standard cycling mode (primer Tm = 60°C). The oligonucleotides used for gene expression analysis were: FOXM1_F: 5′-TCTTTCTTTGTTTATCAGTGCTGC-3′, FOXM1_R: 5′-CCACTTTGATGGGTCTCGCT-3′, MKI67_F: 5′-GGATCGTCCCAGTGGAAGAG-3′, MKI67_R: 5′-CAAACAAGCAGGTGCTGAGG-3′, CCNB1_F: 5′-AACATCTGGATGTGCCCCTG-3′, CCNB1_R: 5′-CTGACTGCTTGCTCTTCCTCA-3′, CENPF_F: 5′-CGTCCCCGAGAGCAAGTTTA-3′, CENPF_R: 5′-GTAGGCAGCCCTTCTTTCCA-3′, H2AFZ_F: 5′-CCAAGACAAAGGCGGTTTCC-3′, H2AFZ_R: 5′-TTTCAGGTGTCGATGAATACGG-3′, PTTG1_F: 5′-ACCCGTGTGGTTGCTAAGG-3′, PTTG1_R: 5′-ACGTGGTGTTGAAACTTGAGAT-3′, HMGB2_F: 5′-CCGGACTCTTCCGTCAATTTC-3′, HMGB2_R: 5′-GTCATAGCGAGCTTTGTCACT-3′, AURKA_F: 5′-GGAATATGCACCACTTGGAACA-3′, AURKA_R: 5′-TAAGACAGGGCATTTGCCAAT-3′, SDH_F: 5′-TGGGAACAAGAGGGCATCTG-3′, SDH_R: 5′-CCACCACTGCATCAAATTCATG-3′

### High-content quantitative Immunofluorescence imaging

MCF-7 cells were plated in 96-well imaging plates (Cellvis, P96-1.5P), transferred to serum reduced and hormone deprived medium and treated with 20 nmol/l β-estradiol for 24h as detailed above. Cells were fixed with 4% PFA/PBS for 10 min, permeabilized for 10 min with ice-cold methanol for 10 min. For EdU staining, the Click-iT reaction was carried out following permeabilization using the ClickiT Cell Reaction Buffer kit (Thermo Fisher Scientific, SC10269) according to the manufacturer's guidelines. The primary antibodies, ERα (Santa Cruz Biotechnologies, sc-8002) and pFOXM1 (Cell Signaling Technology, 14655S) were diluted in 1% BSA-PBS and incubated overnight at 4°C. After three washes with PBS, cells were incubated with secondary antibodies (Invitrogen, A11032, A11034, 1:1000 dilution) and DAPI (5 μg/ml), diluted in 1% BSA-PBS and incubated for 1h at RT. Cells were washed 3x with PBS. Images were captured on an ImageXpress Micro Confocal (Molecular Devices) with a 20× Super Plan Fluor ELWD DM 0.45 NA objective. Fluorescence intensities within a nuclear mask was carried out in MetaXpress (Molecular Devices).

### Multiome analysis

Data were initially processed using *cellranger-arc* (10× Genomics, v2.0.0) using the *count* subcommand with default flags. In total, 6,435 control cells and 7,357 cells with E2 treatment were output. The output was then used to generate a *SeuratObject* using *Seurat* (v4.0.6) in *R* (v4.0.3) (24). For further quality control, transcription start site (TSS) enrichment and nucleosome signal was calculated on the ATAC-seq data using *Signac* (v1.5.0) functions *NucleosomeSignal* and *TSSEnrichment* (39). Data were filtered with the following criteria: ATAC-seq unique reads <100,000 and >500; RNA-seq unique reads <25,000 and >500; nucleosome signal <2; TSS enrichment of >1. 6,023 control (93.6% passing filter) and 7,030 E2 treatment (95.6% passing filter) cells passed the second round of filtering. ATAC-seq peaks were generated using *macs2* (40) called within an *R* session using the function *CallPeaks*, a *Signac* function (39). A count matrix of ATAC-seq reads overlapping peaks was then created using *Signac* functions *FeatureMatrix* and *CreateChromatinAssay*. Cells were then clustered by ATAC profiles using *Seurat* functions *FindTopFeatures*, *RunTFIDF*, *RunSVD* and *RunUMAP* with argument *dims = 2:*40.

Cell lines within sample lanes were then deconvoluted by known copy number variants (CNVs). To do this, *inferCNV (*v1.6.0) was used to calculate copy number variation. *CreateInfercnvObject* was run on the RNA counts matrix generated by *Seurat*. Following this, CNVs were calculated using function *infercnv::run* with arguments *cutoff = 0.1, denoise = TRUE, HMM = FALSE, cluster_references = FALSE*. Output was then plotted and hierarchically clustered via *ComplexHeatmap* with argument *km = 2* (*v2.6.2) (*41). CNV profiles showed little bias between control and treatment conditions. Cell lines were distinguished by plotting UMAP projection overlaid with CNV profile cluster assignment using *Seurat* function *DimPlot*. UMAP projection showed >95% CNV cluster assignment, with UMAP groupings then used to label cell lines. The SeuratObject was then split by CNV profile, and thus cell line, to be processed in parallel.

The RNA counts matrix was processed to generate TITAN topics as described previously. 40 TITAN topics were generated for both MCF-7 and T-47D data sets (Supplementary Table S1). The ATAC counts matrix was used to generate *cisTopic* topics, an orthogonal LDA-based dimensionality reduction using the package *cisTopic (v0.3.0) (*25,26). Topic models for *cisTopic* were generated using the function *runWarpLDAModels* with argument *topic = c (10:30)*. The optimal model was then chosen via *type = derivative* with the function *selectModel*. 20 and 16 cisTopic topics were generated for MCF-7 and T-47D data sets, respectively. Topic models for cisTopic and TITAN were correlated using *base R cor* function and plotted with *ComplexHeatmap*.

Cistrome ChIP-seq data was downloaded and filtered to data sets sourced from MCF-7 or T-47D cell lines, and to those containing at least 1,000 peaks (29–31). Signature enrichment per cell was calculated through *cisTopic* functions *getSignatureRegions* with argument *minOverlap = 0.01*. To calculate motif usage per cell, *ChromVAR (v1.12.0)* was used with motifs from the JASPAR2020 data set (42,43). Motif usages were calculated via *Signac* functions *RunChromVAR*. Accessible regions were linked to promoter sites via the *cicero* package (v1.8.1) via functions *make_cicero_cds, run_cicero, generate_ccans*, and *Signac* function *ConnectionsToLinks* (39,44). UMAP projections were then generated across multiple modalities using *Seurat* and *Signac* functions. RNA UMAP projections were remade per cell line as described previously. ATAC UMAP projections were generated via *FindTopFeatures* with argument *min.cutoff = 5*, *RunTFIDF*, *RunSVD*, and *RunUMAP* with argument *dims = 2:40*. A multimodal projection of RNA PCA and ATAC LSI reductions was performed via *FindMultiModalNeighbors*. UMAP projections of TITAN topics and cisTopic topics were generated with the function *RunUMAP* using all topics. A multimodal projection of both TITAN and cisTopic topics was performed via *FindMultiModalNeighbors*. UMAP projections were then plotted with *DimPlot*.TITAN topics were classified as ‘FOXM1’ or ‘ESR1’ based on which topics had the most gene overlap with the FOXM1 or ESR1 topics defined earlier in their respective cell lines. For MCF-7 cells, TITAN topic 37 was *ESR1* associated, and topic 4 was *FOXM1* associated. For T-47D, TITAN topic 23 was *ESR1* associated, and topic *11* was *FOXM1* associated. A blended feature plot was generated using *Seurat FeaturePlot* with argument *blend = TRUE* (Figure 6B, Supplementary Figure S10A). To test correlation between topics, a linear model was fit with R *base* function *lm*. R^2^ values for fitted lines were 8.235 × 10^−5^ and 0.026. TITAN topic weights were plotted per cell via *ggplot (v3.3.5)* with function *geom_point* and *geom_abline*. Cells were binned for pairwise comparison between cells enriched in *FOXM1* associated and *ESR1* associated topics. This was done by filtering E2 treated cells to those with greater than the 20% quantile for one topic and less than the 75% quantile of the other topic. This resulted in 1,127 and 1,152 MCF-7 cells for *FOXM1* and *ESR1* topics, and 904 and 914 T-47D cells for *FOXM1* and *ESR1* topics, respectively (Figure 6D and Supplementary Figure S10B). Logistic regression was used for pairwise comparison between binned cells for *ChromVAR* deviations, peaks, CistromeDB enrichment, and SCTransform-normalized RNA counts via *Seurat* function *FindMarkers* with additional arguments *latent.vars = ‘nCount_peaks’*. Volcano plots of resulting fold-change and adjusted p values were generated with *ggplot* functions *geom_point* and *geom_text_repel* (from package *ggrepel v0.9.1*, Figure 6E, Supplementary Figure S10D, E).

Additional joint-embedding strategies were performed for comparison to our topic modeling method. We used scREG (45), a reduction method focused on cis-regulatory networks between peak-gene pairs, and Amateur (46,47), a deep learning methodology using an autoencoder to define a reduced space of latent features (Supplementary Figure S10F). For scREG (v0.1.0), we modified data input functions to suit our preexisting Seurat Objects. Following dimensionality reduction with the *RegNMF* function using default arguments, we integrated the output data frame into Seurat and used *Seurat* functions *RunUMAP* on RegNMF output with *dims = 1:100*. For Amateur, we first generated cell cycle scores using the *Seurat* function, *CellCycleScoring* using the default gene set. We modified an output function to write out our preexisting Seurat Objects in a format compatible with Amateur and processed our data as suggested. Briefly, H5AD formatted matrices were generated, data was *L1* normalized and *singular value decomposition* SVD matrices were generated via *sklearn* function *TruncatedSVD*. Amateur is a semi-supervised algorithm which accounts for cell labels and cell cycle scores during training, to this end we supplied annotations of *cell_type_labels = mod1_obs[‘topic_bin’]* (our previously defined binned cells by topics), *batch_ids = mod1_obs[‘sample’]* (our batch information), and *phase_labels = mod1_obs[‘Phase’]* (from our Seurat package *CellCycleScoring*). The autoencoder was built as previously described (47), and the *joint.embed* output, consisting of 12 latent features, were added to our Seurat objects and *Seurat* function *RunUMAP* with argument *dims = 1:12*, was used for final dimensionality reduction.UMAP reductions were plotted with *FeaturePlot* and *DimPlot* functions as described above. We found these processes to have roughly equivalent ability to separate out FOXM1 and ESR1 topics to our topic-modeling method, despite their orthogonal approaches.

Coverage plots across genomic locations were generated using the *Signac* function *CoveragePlot* with arguments *expression.assay = ‘SCT’* (Figure 6F, Supplementary Figure S10G, H). Genes *PGR* and *CENPF* were chosen as both were differentially expressed between *FOXM1* and *ESR1* binned cells and had > 3 overlapping peaks with nominally significant differential accessibility. Peaks were plotted along genomic tracks. Peaks with a DNA sequence matching the ESR1 motif or FOXM1 motif were colored using *motifmatchr* (*v1.12.0*) for classification. Cis-correlation linkage between peaks calculated using cicero (described above) were plotted. Peaks which showed at least nominal significance (*P*-value < 0.05) were highlighted in a grey box.

## RESULTS

### Quantifying estrogen response pathways using TITAN

**Figure 1.**
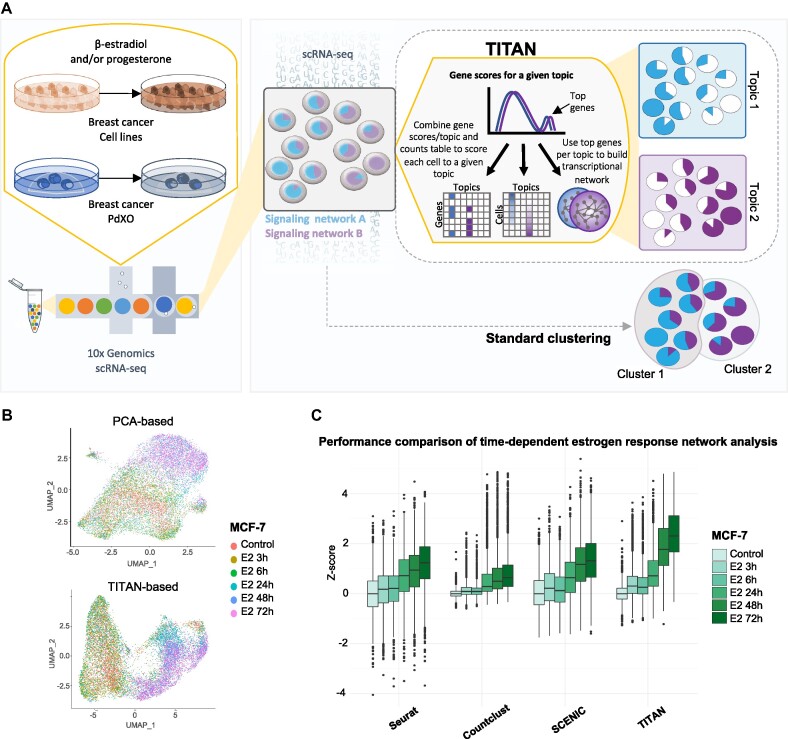
TITAN, a topic modeling approach to scRNA-seq analysis, identifies distinct gene sets (topics) specific to estrogen signaling gradients. (**A**) Schematic representation of experimental setup and TITAN: Topic Inference of Transcriptionally Associated Networks in scRNA-seq. Breast cancer cell and PdXO models are subject to 100 nmol/l β-estradiol and/or 100 nmol/l progesterone stimulation, triggering signaling network changes across cells. Using Latent Dirichlet Allocation (LDA), TITAN identifies latent transcriptional networks, or topics, in the scRNA-seq dataset. Topics are linked by a distribution of scores that relate genes to topics and topics to cells. These scores are then used to infer transcriptional networks. TITAN identifies distinct gene gradients in comparison to standard clustering approaches which are binary distributions. (**B**) PCA-based (top) and TITAN-based (bottom) UMAP visualization of MCF-7 cells treated with estrogen for different time points. (**C**) Comparison of *Z*-scores normalized to control in time-dependent estrogen response networks for TITAN, PCA-based clustering (Seurat), SCENIC and CountClust.

We investigated whether response to hormones in a synchronized cell population is uniform or heterogeneous. To this end, we performed scRNA-seq on four ER positive (ER+) cell lines, MCF-7, T-47D, ZR-75–1 and the tamoxifen resistant MCF-7 cell line, TAMR, as well as two ER+ patient-derived explant organoids (PdXO), HCI003 and HCI011 (48). Cells were conditioned in hormone deprived and reduced serum media for three days before stimulation with β-estradiol (E2) and/or progesterone (PG) for 0, 3, 6, 24, 48 or 72 hours (h) (Figure 1A). To aid in mapping signaling gradient changes in response to our treatment, we developed TITAN. TITAN infers gene expression gradients or ‘topics’ in the system and assigns a cell-topic score for every single cell, as well as a gene-topic score for every gene within a topic (Figure 1A). The gene sets that constitute each topic can then be analyzed for pathway enrichment using available pathway analysis methods such as Enrichr (34,35).

As an initial benchmarking experiment for the method, we compared cell lineages identified in a single-cell peripheral blood mononuclear cell (PBMC) dataset (10x genomics) using PCA based UMAP/Louvain clustering (49) and TITAN. Supplementary Figure S1A shows the number of topics generated for all datasets in this study as described in methods. We observed a need for higher topic numbers in the highly heterogeneous PBMC and primary tumor datasets in comparison to the cell line datasets, expected to be relatively homogenous. For the PBMC dataset, 50 topics generated by TITAN were able to show distinct transcriptional patterns in major cell clusters identified by known markers, in a manner analogous to the PCA-based approach (Supplementary Figure S1A–C). Although separating well-defined lineages is not TITAN’s primary purpose, this analysis indicates that TITAN correctly identifies variable gene sets required to differentiate major lineages independent of traditional clustering methods. The list of top 50 genes per topic of all datasets are listed in Supplementary Table S1.

To evaluate TITAN’s performance in uncovering signaling gradients in scRNA-seq data specifically, we analyzed ER+ cell lines treated with estrogen alone. Our scRNA-seq of MCF-7, T-47D and ZR-75-1 resulted in a total of 14 796, 10 424 and 9433 cells respectively. We performed the standard PCA based dimensionality reduction and UMAP cluster visualization, revealing seven, four and six transcriptional clusters in the MCF-7, T-47D and ZR-75–1 cell lines respectively (Supplementary Figure S2A). We observed that while some groups of cells form distinct clusters on the UMAP, separation between clusters was less defined overall. To assess the impact of hormone signaling on cluster definition, we plotted the proportion of cells in each cluster across time. Remarkably, we observed that underlying heterogeneity in the system was influenced by hormone treatment as certain cluster sizes increased, decreased or stayed the same with estrogen signaling (Supplementary Figure S2B). This suggests that transcriptional heterogeneity observed in these cancer cell lines exhibits a level of plasticity and responsiveness to estrogen.

Figure 1B shows the difference between UMAPs generated using standard PCA or TITAN inputs, with TITAN showing slightly improved separation of earlier and later time points. We further compared TITAN’s performance to two other tools used for gene regulatory network (GRN) analysis; SCENIC (33) and CountClust (34). Estrogen signaling pathways are expected to increase over time with estrogen treatment, and this ground truth knowledge enhances tool comparison accuracy. The top estrogen upregulated topic identified by TITAN was compared against clusters or networks identified as being driven by estrogen in the other methods (see methods and Supplementary Figure S3A-B) and a *Z*-score analysis was performed. Results indicated that all four approaches captured response to estrogen across time, however TITAN showed the highest *Z*-score enrichment indicating its ability to better capture dynamic changes in the estrogen signaling pathway (Figure 1C, Supplementary Figure S3D). While SCENIC was able to identify several transcriptional networks such as GRHL2, GRHL3 and HES1 changing in a treatment specific manner, the expected ESR1 regulon was not identified as significantly changed across time or treatments (Supplementary Figure S3C). Lastly, we compared CPU time and RAM usage for all tools run on the same data, with TITAN requiring the least resources and time (Supplementary Figure S3E).

### Divergent transcriptional networks activated in response to estrogen

**Figure 2.**
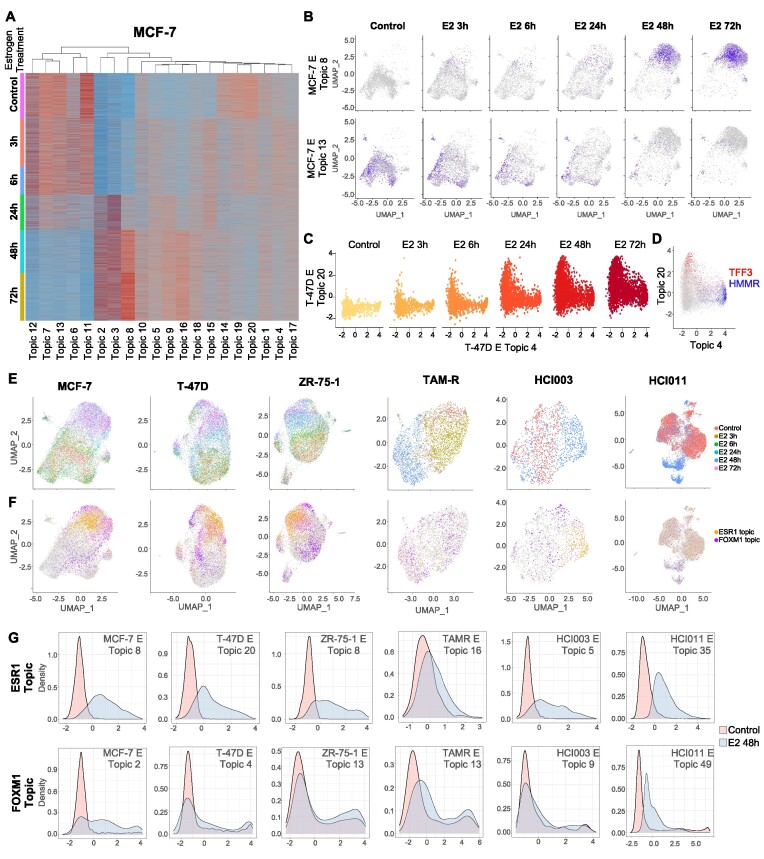
Divergent estrogen driven topics identified in breast cancer cell lines and organoids. (**A**) Heatmap of TITAN normalized cell topic scores for MCF-7 cells treated with estrogen for different time points. (**B**) Feature plot representation of Topic 8, representative of topics increased by longer estrogen treatments and Topic 13, representative of topics decreasing with longer estrogen treatments. (**C**) Scatter plot representation of T-47D cells expressing topic 20 and topic 4 per duration of estrogen treatment. (**D**) Scatter plot representation of topic 20, with expression levels of TFF3 (red) and topic 4 with expression levels of HMMR (blue), genes associated specifically with each respective topic. (**E**) UMAP dimensionality reduction visualization of MCF-7 (14,788 cells), T-47D (10,424 cells), ZR-75–1 (9,433 cells), TAMR (3,750 cells), HCI003 (1,615 cells) and HCI011 (13,470 cells) breast cancer models colored by estrogen treatment duration (**F**) UMAP dimensionality reduction visualization of the same cells as (E) by expression of FOXM1 (purple) and ESR1 (orange) driven topics. (**G**) Ridge-plot representation of ESR1 (red) and FOXM1 (blue) topic expression distribution in breast cancer models treated with estrogen for 48 h.

To better model inter-tumor heterogeneity in estrogen signaling, we expanded our study to four cell lines and two PdXOs treated with estrogen (Figure 2A, E, Supplementary Figure S2A–C and S4A). In MCF-7 cells, several topics were observed to increase or decrease in response to estrogen with differing dynamics. To identify pathways associated with a given topic, we selected the top 50 genes in a given topic based on gene distribution histograms (Supplementary Figure S2D, Table S1) and performed Enrichr (35–37). We first focused on the highest estrogen up- and down-regulated topics, topic 8 and 13, respectively (Figure 2B). Pathway analysis of these topics confirmed the expected ER and FOXA1 (50) pathways to be upregulated with treatment in topic 8, while known estrogen downregulated pathways such as TGFB and MYC (51,52) were enriched for topic 13 (Supplementary Figure S2E).

However, in addition to topic 8 and 13, there existed multiple other estrogen up- or down-regulated topics. We compared topics in MCF-7, T-47D and ZR-75–1 against each other by plotting their cell-topic scores to identify dissimilar topics. Several estrogen responsive topics were identified to be divergent from each other and had differing rates of response to ligand (Supplementary Figure S5A, B). Topics 20 and 4 in the T-47D cell line, indicated that while both were increased by estrogen, the two topics were activated in distinct sets of cells (Figure 2C) and driven by markedly different genes (Figure 2D). Enrichr pathway analysis showed topic 4 to be driven by the transcription factor FOXM1, while topic 20 was enriched for ER. FOXM1 has been previously described to be important in regulating ER expression and also to co-bind with the receptor based on ChIP-sequencing assays (53,54).

As an orthogonal comparison to the Enrichr analysis, we tested the significance of overlap between genes in all 20 topics of our TITAN analysis and the CistromeDB (31) database of ChIP-seq studies performed in MCF-7, T-47D and ZR-75–1 (1,160 datasets) (see Materials and Methods and Supplementary Table S2). Results again indicated that not all estrogen induced topics were enriched for ER binding sites (Supplementary Table S2). Topics 20 and 4 in T-47D showed enrichment for ER and FOXM1 respectively, consistent with our Enrichr analysis.

We then investigated the occurrence of divergent ER and FOXM1 driven topics in the other models. We generated a table representing the gene overlap between all topics for all the models treated with estrogen (Supplementary Table S3). In all datasets, we found topics that correlated with the FOXM1 and ESR1 topics described in MCF-7. We confirmed their dependence on the relevant transcription factor by Enrichr analysis (Supplementary Tables S2 and S3). We projected the cells enriched for either topic on the standard UMAP and observed them occupying distinct UMAP spaces (Figure 2E, F). Distribution plot analysis of the ESR1 and FOXM1 topics confirmed that both topics increased with estrogen treatment and were present in all models (Figure 2G). Additionally, we were able to identify organoid specific topics like Topic 29 for HCI003 and topics 8, 29, 40 and 47 for HCI011 that encompass genes involved in various cell signaling pathways dependent on interferon and histone lysine demethylase activity.

To determine whether the heterogeneous response to estrogen is due to the presence of genetically distinct subclones, we performed scRNA-seq in two clonally derived MCF-7 cell lines after treatment with estrogen for 24 or 48 h. When compared to the above non-clonal scRNA-seq datasets at matched time points, results indicated that cells from both clones exist in all single-cell clusters (Supplementary Figure S6A, B), showing that PCA based UMAP clustering is not driven by genetic clones. We performed TITAN analysis and observed estrogen driven topics in all datasets, albeit with varying dynamics (Supplementary Figure S6C). Once more, both clone 1 and clone 2 demonstrated simultaneous presence of topics associated strongly with ER (topic 8) and the FOXM1 derived topic 2. Unlike standard clustering methods, gene sets in each topic identified by TITAN can be transferred onto different datasets (see methods). Hence, to confirm the presence of the original topics in MCF-7 cells, we transferred the expression of the top 50 genes in estrogen treatment topics from the MCF-7 mixed population data on to the clonal population data (Supplementary Figure S6D) and found both the FOXM1 topic (topic 2) and the ESR1 topic (topic 8) to be upregulated upon estrogen in both clones. The TITAN analysis hence indicates that alternate ER or FOXM1 linked responses are not clonally derived but rather suggestive of plastic or oscillatory states within the population.

### Estrogen and progesterone can activate both shared and unique topic signatures

**Figure 3.**
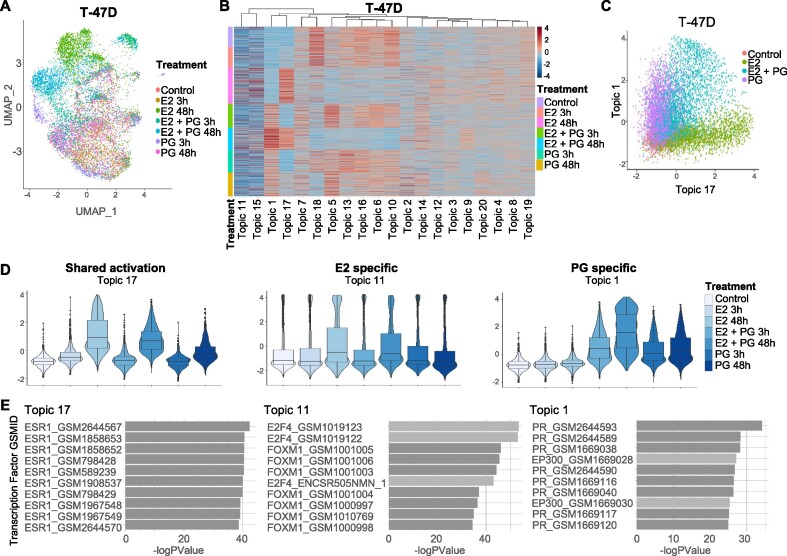
Estrogen and progesterone induce both unique and shared gene expression signatures. (**A**) PCA based UMAP dimensionality reduction of T-47D (10,668 cells) treated with estrogen (E2), progesterone (PG) alone or in combination (E2 + PG) for 3 and 48 h, colored by treatment. (**B**) Heatmap visualization of TITAN normalized cell topic scores for T-47D of the same dataset. (**C**) Scatter plot representation of topic 1 (y-axis) which represents networks regulated by progesterone treatment compared to topic 17 (x-axis) which represents a shared gene set activated by both hormones colored by type of treatment. (**D**) Violin plot representation of topic 17 (left) representative of gene sets activated by both hormones, 11 (center) representative of gene sets activated primarily by estrogen and 1 (right), representative of gene sets activated by progesterone. (**E**) Transcription factor (TF) enrichment analysis of genes involved in topics 17 (left), 11 (center) and 1 (right) using publicly available ChIP datasets from CistromeDB (29,30), significance of overlap *P*-values is generated by hypergeometric test.

In addition to the estrogen signaling pathway, progesterone and its receptor (PR) expression is a major determinant of disease outcome in breast cancer (55) and PR has been shown to exhibit direct crosstalk with ER (3,4). Activation of PR by progesterone is known to reprogram ER genomic binding patterns and elicit unique downstream signaling events. However, these roles are presumed to be highly context specific and the interplay between these networks is not known at a single-cell level. Delineating the two pathways is made harder by the substantial overlap in downstream genes that they regulate. In order to evaluate whether TITAN can infer gene expression changes under multiple signaling cues, we performed scRNA-seq experiments after stimulation with estrogen, progesterone or both hormones for 3 or 48 h. Experiments were performed in MCF-7, T-47D and ZR-75–1 cell lines after hormone deprivation. The three cell lines have been shown to express different levels of ER and PR and hence, their dependence on these pathways is likely to be different (56). PCA based dimensionality reduction and UMAP cluster visualization indicated the presence of several clusters for all three cell lines (Figure 3A, Supplementary Figure S7A). These clusters were poorly defined and treatment groups were spread across all clusters. Similar to the previous estrogen only treatment experiments, the proportion of cells in each cluster were seen to exhibit shifts in response to both progesterone and estrogen treatments (Supplementary Figure S7B). TITAN analysis in the dual hormone experiment identified groups of genes observed to increase or decrease in expression with treatment conditions (Figure 3B, Supplementary Figure S7C). As an example, in T-47D cells, plotting the cell-topic scores for topic 1 and 17 resolved treatment specific transcriptional differences and found genes otherwise undetected in standard clustering and differential expression (Figure 3C, Supplementary Table S1). Using TITAN, we observed topics unique to either the progesterone or estrogen treatment such as topic 1 and 11 respectively (Figure 3D). Further, gene sets such as those in topic 17 were seen to be upregulated by both hormones separately or in combination. These results identify the presence of distinct gradients of gene expression networks unique to each hormone and others shared between the two.

Given that both ER and PR are transcription factors that directly activate or repress their respective target genes, we investigated overlap between topics and the CistromeDB database as described above. Results indicated that topic 17, which was observed to be activated by both progesterone and estrogen was enriched for ER binding patterns, while progesterone specific topics such as topic 1 was enriched for PR binding patterns (Figure 3E). Interestingly, the estrogen unique topic 11 showed enrichment for FOXM1 and E2F4 and not ESR1, allowing us to better define shared or unique hormone dependent gene signatures. This observation was also noted for topic 6 for MCF-7 and topic 12 for ZR-75-1 cells (Supplementary Figure S7D).

### Phosphorylation of FOXM1 in response to estrogen activates distinct gene signature

**Figure 4.**
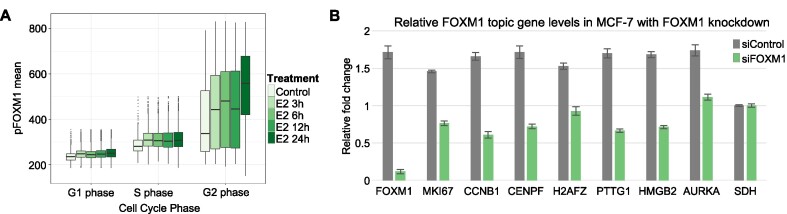
FOXM1 is activated through phosphorylation and regulates corresponding topics upon estrogen stimulation. (**A**) Distribution of pFOXM1 protein levels quantified as mean fluorescence intensity within 37,911 nuclei in MCF-7 cells plotted by cell cycle phase and estrogen (20 nmol/l) treatment length imaged by high-content quantitative imaging. Boxplot hinges correspond to the 25th–75th and whiskers correspond to 1.5 × IQR (inter-quartile range) of the hinge. Outlying points are plotted individually. (**B**) Relative gene expression of FOXM1 targets in MCF-7 cells with 20 nmol/l estrogen treatment for 6 h. SDH gene used as internal control. Error bars represent s.e.m. in three biological replicates.

Our topic analysis indicated the presence of distinct ER and FOXM1 driven gene expression networks in response to estrogen stimulation. We then aimed to functionally validate the role of FOXM1 in activating these gene sets. In addition to being an ER interactor, we and others have shown FOXM1 to be important in cell cycle regulation and exit from S-phase coincident with phosphorylation at Thr600 (57,58). We thus investigated whether FOXM1 is activated by phosphorylation of Thr600 in response to estrogen. Estrogen treated MCF-7 cells were stained with pFOXM1 and ERα antibodies as well as EdU for cell cycle phase assignment (Supplementary Figure S8A). Stimulation of MCF-7 cells by estrogen increased Thr600 phosphorylation of FOXM1 particularly in the G2 phase of the cell cycle relative to control (Figure 4A). ERα expression decreased with estrogen treatment as previously observed (59,60) in a cell cycle independent manner (Supplementary Figure S8B).

To confirm the FOXM1 dependent regulation of genes identified in the FOXM1 topic, we silenced FOXM1 using siRNA in MCF-7 cells treated with estrogen (Figure 4B). All tested genes from the FOXM1 topic were observed to increase in expression with estrogen and the baseline expressions of these genes were attenuated when FOXM1 was knocked-down (Supplementary Figure S8C). This suggests that the genes in this topic are indeed regulated by FOXM1 in response to estrogen.

### Distinct expression of FOXM1 and ESR1 topics in primary breast cancer

**Figure 5.**
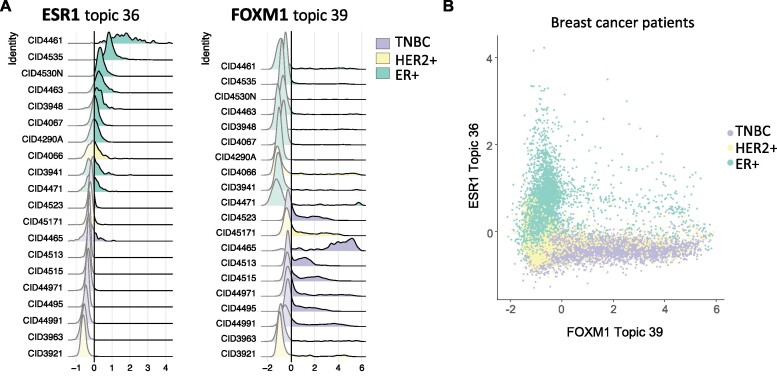
Divergent ESR1 and FOXM1 topics identified in breast cancer patient scRNA-seq datasets and show subtype preference. (**A**) Ridge-plot representation of ESR1 (left) and FOXM1 (right) topic expression distribution in 36,798 epithelial cells from 20 breast cancer patients colored by tumor subtype. (**B**) Scatter plot representation of the ESR1 topic 36 (y axis) compared to the FOXM1 topic 39 (x axis) colored by tumor subtype in 20 breast cancer samples. Data used in this figure are from Wu *et al.* (13).

To further understand the role of ER and FOXM1 linked topics identified in cell lines, we applied TITAN on a previously published patient breast tumor scRNA-seq dataset (13). Wu *et al.* performed scRNA-seq on 26 primary tumors of different subtypes. We analyzed the 36,798 epithelial cells defined in their research using TITAN. It is worth noting that subtype clusters were qualitatively better separated in the TITAN based UMAP space compared to the PCA based UMAP (Supplementary Figure S9A). The TITAN heatmap revealed substantial intra- and inter-tumor topic heterogeneity with some topics differentiating between samples or subtypes and others being common in all samples (Supplementary Figure S9B). For example, topics 8, 21, 32 were expressed across all subtypes while topics 23 and 36 were only seen in ER+ samples. Topic 29 on the other hand was present in all HER2+ samples while being most highly expressed in the HER2+ patient CID45171. Enrichr analysis of TITAN results indicated that topic 36 corresponded to the ESR1 topic and topic 39 corresponded to the FOXM1 topic. The genes in topic 36 and topic 39 were also observed to be the most correlated with the ESR1 and FOXM1 topics in our cell line models respectively (data not shown). We then ordered the patient samples by level of ESR1 topic expression and observed that ER+ patients had the most ESR1 topic expression as expected. Interestingly however, samples low in ESR1 topic showed a high FOXM1 topic expression and predominantly consisted of triple negative breast cancer (TNBC) samples (Figure 5A). When we plotted cell topic scores of ESR1 topic and FOXM1 topic, we observed that cells expressed the two topics in distinct sets of cells primarily in a subtype specific manner (Figure 5B). Thus, we show that our findings from the cell line models align with patient datasets.

We further explored intra-patient heterogeneity of transcriptional cell states using a separate dataset (9) consisting of an ER + luminal B type primary tumor (BC03) and matched lymph-node metastasis (BC03LN). We transferred ESR1 and FOXM1 topics identified in the T-47D cell line onto these patient datasets. In the primary tissue, we observed higher ESR1 topic (topic 17) and reduced FOXM1 topic (topic 11) (Supplementary Figure S9C). Interestingly, for the matched lymph node metastasis, the opposite trend was observed with decreased ESR1 topic and increased FOXM1 topic, showcasing transcriptional plasticity within a patient. It is noteworthy that overexpression of FOXM1 has been described to be strongly associated with lymph node metastasis in breast cancer (61) and hence supports our analysis. Correspondingly, we observed increased ER gene expression in the primary tumor while FOXM1 gene expression was increased in the metastatic sample (Supplementary Figure S9D). Kaplan-Meier analysis of the top 50 genes driving the ESR1 topic (topic 17), indicated that higher expression of the ESR1 linked topic genes correlated with better patient outcome, while on the contrary, the top 50 genes driving the FOXM1 topic (topic 11) indicated significantly poorer patient outcome, suggesting distinct clinical roles for these two topics (Supplementary Figure S9E).

### Estrogen stimulation directs transcriptionally and epigenetically distinct cell states

**Figure 6.**
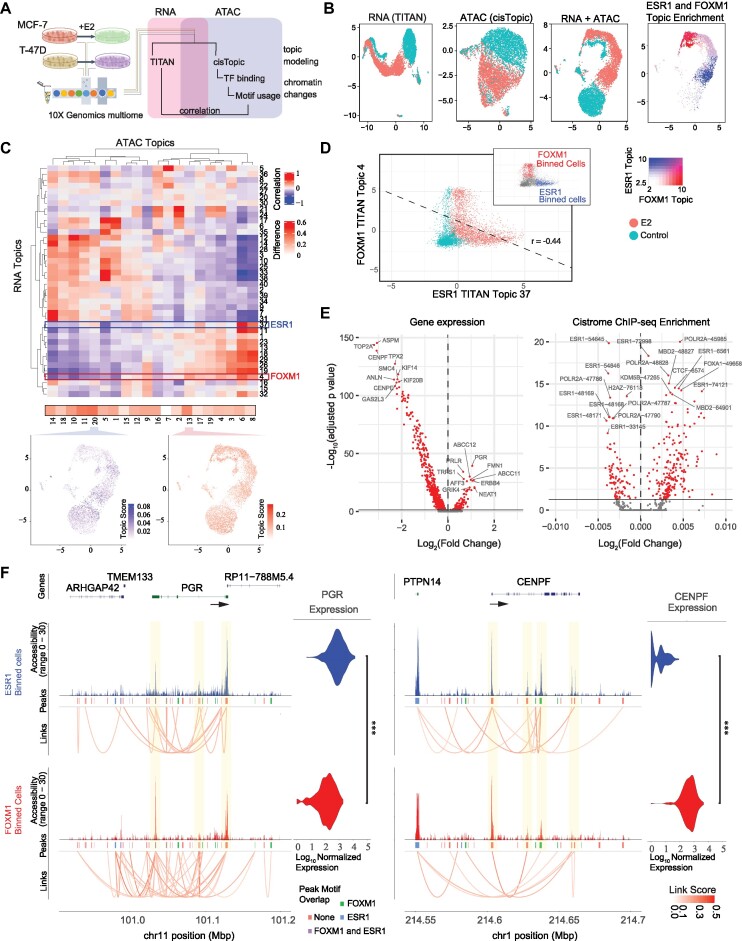
Multiomic analysis reveals divergent gene expression and chromatin states upon estrogen stimulation. (**A**) Schematic of experimental design and analysis of multiome single cell profiles in MCF-7 and T-47D cell lines with and without 20 nmol/l β-estradiol (E2) treatment. RNA profiles undergo TITAN topic modeling and paired ATAC profiles undergo cisTopic topic modeling. Results are then correlated. (**B**) UMAP projection of MCF-7 (left, 6,438 cells) cell profiles colored by treatment. Projections were calculated using TITAN, cisTopic, and TITAN + cisTopic profiles (see Methods). UMAP projection of TITAN + cisTopic profiles colored by ESR1 and FOXM1 defined TITAN topics (far right). Color legend shared with panel (D). (**C**) Topic expression heatmap correlation between TITAN and cisTopic topics. TITAN ESR1 (Topic 37, blue rectangle) and FOXM1 (Topic 4, red rectangle) topics are highlighted. UMAP projection of TITAN + cisTopic profiles colored by cisTopic topics 20 (left) and 13 (right) showing open chromatin regions specific to TITAN topics (bottom). (**D**) Scatterplot of FOXM1 and ESR1 TITAN topic values in MCF-7. Main figures are colored by treatment with R2 correlation and linear fit shown as a black line. Inset panels show binned FOXM1 and ESR1 topic enriched cells. These bins were defined as cells having ≥20% quantile value for the respective bin and ≤75% quantile value of the opposite bin. This resulted in 1127 and 1,152 MCF-7 cells for FOXM1 and ESR1 topics respectively. Color legend shared with panel (C). (**E**) Volcano plot of differential expression and cistrome defined enrichment scores of ChIP-seq peaks between FOXM1 (right) and ESR1 (left) topics for binned MCF-7 cells defined in panel (D). The top 10 genes or CistromeDB peak sets are shown for FOXM1 and ESR1 bins. Names displayed show genes or target protein and cistrome ID. Positive Log2 fold-change reflects ESR1 bin enrichment. Points plotted in red are significant (adjusted *P*-value ≤ 0.05). (**F**) Coverage plot of aggregate ATAC profiles for binned MCF-7 cells defined in panel (D). Genome track covers the gene body of PGR (left) and CENPF (right) with 5kbp up and downstream. Black arrow represents the transcription direction. Side plot displays the differential gene expression of PGR. Peaks subpanel shows defined open regions on aggregate data, peaks are then colored by overlap with FOXM1 and ESR1 binding motifs. Links subpanel displays cis-coaccessibility linked between peaks, displaying peaks which are correlated. Gray highlight boxes overlap peaks which show at least nominal significance (logistic regression, *P*-value ≤ 0.05). *** denotes adjusted logistic regression *P*-value ≤ 0.01.

To investigate whether different transcriptional signatures in response to estrogen are a consequence of distinct chromatin states, we performed single-cell multiome sequencing, an assay that simultaneously analyzes mRNA expression and chromatin accessibility (ATAC-seq) from the same cell. MCF-7 and T-47D cells were subject to vehicle control or 48h estrogen treatment (Figure 6A) and single-cell multi-ome analysis was performed. Resulting single-cell transcriptomes from the dataset were analyzed using TITAN and chromatin states were identified using cisTopic, also an LDA based topic modeling approach developed for scATAC-seq analysis. UMAPs were generated separately using TITAN (RNA), cisTopic (ATAC-seq) and both modalities combined (Figure 6B, Supplementary Figure S10A). Treatment based UMAP separations were observed for the MCF-7 cell line, while these differences were less distinct in the T-47D cell line. Once again, distinct ESR1 and FOXM1 topics were identified in both datasets by TITAN and were observed to occupy separate UMAP spaces in both cell lines (far right panels of Figure 6B and Supplementary Figure S10A, B) suggesting distinct chromatin and transcriptional states for these cells. The topic modeling-based frameworks for both TITAN and cisTopic allowed us to directly correlate transcriptional and chromatin accessibility-based patterns. Results identified differentially accessible regions associated with ESR1 and FOXM1 RNA topics (Figure 6C and Supplementary Figure S10C). While some ATAC topics overlapped with both ESR1 and FOXM1 RNA topics, chromatin patterns unique to FOXM1 or ESR1 were also observed, with cisTopic topics 20 and 13 highlighted as examples in MCF-7. Again, examples highlighted in both cell lines show that these topics occupy different UMAP spaces. To further resolve chromatin level differences between the two RNA topics, we selected cells enriched for ESR1 or FOXM1 signatures. We plotted expression levels of the FOXM1 and ESR1 TITAN topics and confirmed that these two topics are enriched in an anti-correlative manner in distinct cells (Figure 6D, Supplementary Figure S10B). The top 20% of cells enriched in either topic (see methods) was selected and we performed differential gene expression, transcription factor enrichment using the CistromeDB ChIP-seq datasets and transcription factor-associated accessibility analysis using ChromVAR (42) (Figure 6E, Supplementary Figure S10D, E, Table S4). To ensure regulatory differences between topics segregated without *a priori* knowledge of topics, we applied two orthogonal ATAC + RNA joint embedding dimensionality approaches, scREG (45) and Amateur (46,47). Both analyses demonstrate the same clear separation of cell states enriched for FOXM1 and ESR1 topics (Supplementary Figure S10F). Differential gene expression results identified substantial differences between the two groups with classical ER response genes such as PGR and TRPS1 being enriched for the ER topic while known FOXM1 targets such as CENPE and TOP2A (62) being enriched for its corresponding topic. For the transcription factor enrichment analysis, ESR1 came up as an enriched transcription factor for both topics which is not surprising since FOXM1 is known to bind to chromatin at non-consensus sequences through cofactors such as ER (53,54,63). This characteristic of FOXM1 also results in ChromVAR analysis outputting cofactor related TF motifs.

We next looked at the ATAC accessibility profiles of top genes from both topics (Figure 6F, Supplementary Figure S10G, H). The cells in the ESR1 topic have a significantly higher PGR expression compared to the cells in the FOXM1 topic. As a result, the transcription start site (TSS) of PGR (highlighted) in cells from the FOXM1 high group is less accessible than the same promoter region in cells from the ESR1 high group. The opposite can be stated for the TSS of the CENPF gene which has a higher expression in the cells from the FOXM1 topic bin. Other differentially accessible regions are present on the gene body and transcription termination sites (TTS) indicating that these genes are differentially regulated in cells highly expressing one or the other topic. We further investigated chromatin states at these genes using Cicero, a tool that identifies patterns of shared accessibility. Results identified substantial differences between patterns of co-accessibility between the two topic groups, suggesting chromatin state differences between the two topics.

## DISCUSSION

5–20% of ER+ breast tumors show intrinsic resistance to endocrine therapy and over-time, 30–40% of patients will acquire this resistance (64,65). Distinct transcriptional or mutational cell states contribute greatly to this variable response to therapy (14,66,67). Understanding disease evolution requires us to establish fundamental variability in hormone response pathways. Single-cell sequencing technologies need to be coupled to the development of computational analysis methods in order to efficiently pave the way in defining novel cell-states and transcriptional networks. Here, we provide a comprehensive study characterizing transcriptional and chromatin changes in response to estrogen stimulation in breast cancer models at single-cell resolution. To accurately assess these changes, we first developed a novel analysis tool, TITAN, to detect and quantify signaling gradients in scRNA-seq datasets and showed better performance in comparison to the other tools tested. The method does not rely on discrete classifications of cells into cluster groups, allowing better capture of transcriptional gradients. Furthermore, TITAN does not require prior knowledge about the system, allowing the discovery of new transcriptional networks. Key to TITAN’s success is the CLR based normalization approach, without which, the dataset is strongly influenced by highly abundant genes such as ribosomal and mitochondrial genes. Our results analyzed with CountClust (34), also an LDA based approach, showed high enrichment for such genes (e.g. Clusters 1 and 4, Supplementary Table S1), highlighting the importance of the normalization approach. A limitation of the TITAN method is that it does not have the function to predict probable master regulators that drive the genes in a given topic, requiring us to couple our results to CistromeDB TF enrichment (29–31) and Enrichr pathway analysis (34,35), a feature that could be incorporated in the future. Heterogeneity is a product of multiple regulatory cell mechanisms coming into play at a certain time, in a certain environment. Defining cell states that reflect this heterogeneity is greatly improved by combining data from multiple layers of transcriptional regulation analyzed in a complementary manner. TITAN and cisTOPIC (26) are easily coupled to analyze multiomic data from gene expression and chromatin accessibility profiles in cell lines treated with estrogen. Supplementing our RNA expression analysis with nucleosome accessibility data allowed further stratification of TITAN defined cell states.

Using TITAN, we discovered multiple estrogen driven topic groups changing across time. We focused on two estrogen upregulated topics that seem to be active in non-overlapping sets of cells and were predicted to be driven by ER and FOXM1 in all cell line and organoid models tested. The two cell groups demonstrated markedly different chromatin states indicating that these transient cell states are tightly regulated at the chromatin and transcription factor level. We and others have characterized FOXM1’s role in cell cycle regulation and hence increased FOXM1 and associated cell cycle activity is not unexpected with estrogen stimulation. However, it is key to note that this FOXM1 state is associated with suppression of classical ER responsive genes such as the progesterone receptor, creating the potential for an oscillatory system. Given that FOXM1 is not a hormone receptor, its activity in response to estrogen is likely to be influenced by ER. The two factors are co-bound to chromatin in over 80% of FOXM1-bound regions in ER + cell lines while having an altered binding pattern in TNBC cell lines (53). Beyond crosstalk at the chromatin level, FOXM1 has been reported to both regulate ERα expression (54) and be itself regulated by both isoforms of ER (ERα and ERβ) (68,69), further highlighting the interplay between two factors. Interestingly, we show that unlike ER, PR is unable to activate the FOXM1 network. This may be due to the hormone having anti-proliferative effects in the context of ER positive disease, as shown by us and others (3,4,70). Other studies suggest a proliferative role for progesterone (71). These differences are likely driven by context specific differences such as cell cycle phase or coregulation by other nuclear receptors (72,73).

So why does this heterogeneity matter from a clinical perspective? By current recommendations, 1% ER expression in tumor histology is sufficient for the tissue to be assigned as ER positive (74). Interestingly, it has been shown that tumors with ER positivity as low as 10% can show significant response to anti-estrogen therapy (75–77). This observation has intrigued clinicians and researchers alike over the decades. It is plausible that a greater percentage of cells are ER driven in the system, but cycle through ER high and low cell states, hence an IHC based snapshot would underestimate ER activity in such a system. Cell cycle dependent oscillations in ER levels have been reported and suggested to be a mechanism of resistance to endocrine therapy (78). Similarly, we observe variations in ER topic expression across cell cycle states. Endocrine resistant cell line models that we tested showed increased dependence on the FOXM1 state over the ER, a finding consistent with our patient-based results comparing primary and lymph node metastases (Figure 5, Supplementary Figure S9) and existing studies of FOXM1 expression being linked to lymph node metastasis (61). This reinforces the notion that in addition to targeting ER, drugging potential routes to resistance such as FOXM1 holds merit. A recent study demonstrated the promise of FOXM1 targeting compounds for suppressing breast tumors in preclinical models (62).

TITAN has been valuable in uncovering novel pathways in our breast cancer datasets and we envision its application in additional cancers and other biological contexts where signaling heterogeneity gradients runs independent of traditional clusters.

Our systematic analysis of hormone states has, for the first time, shown the extent of transcriptional and epigenetic heterogeneity induced by estrogen. These results will help identify precise changes as tumors evolve, aiding in better diagnosis and treatment of the disease.

## DATA AVAILABILITY

The scRNA-seq data on breast cancer cell lines is available under the accession number GSE154873. The package can be accessed at https://github.com/ohsu-cedar-comp-hub/TITAN.

Code for figures and analysis not related to the TITAN pipeline can be accessed at https://github.com/ohsu-cedar-comp-hub/BC_estrogen_TITAN_paper2022.

Source data files for PBMC scRNA-seq data are available on the 10x genomics website as ‘3k PBMCs from a Healthy Donor’ at https://www.10xgenomics.com/resources/datasets/.

The breast tumor scRNA-seq datasets were downloaded from NCBI Gene Expression Omnibus database with the accession numbers GSE75688 (9) and GSE176078 (13).

## Supplementary Material

gkac908_Supplemental_Files

## References

[1] Shang Y. , Hu X., DiRenzo J., Lazar M.A., Brown M. Cofactor dynamics and sufficiency in estrogen receptor–regulated transcription. Cell. 2000; 103:843–852. 11136970 10.1016/s0092-8674(00)00188-4

[2] Métivier R. , Penot G., Hübner M.R., Reid G., Brand H., Koš M., Gannon F. Estrogen receptor-α directs ordered, cyclical, and combinatorial recruitment of cofactors on a natural target promoter. Cell. 2003; 115:751–763. 14675539 10.1016/s0092-8674(03)00934-6

[3] Mohammed H. , Russell I.A., Stark R., Rueda O.M., Hickey T.E., Tarulli G.A., Serandour A.A., Serandour A.A.A., Birrell S.N., Bruna A. et al. Progesterone receptor modulates ERα action in breast cancer. Nature. 2015; 523:313–317. 26153859 10.1038/nature14583 PMC4650274

[4] Singhal H. , Greene M.E., Tarulli G., Zarnke A.L., Bourgo R.J., Laine M., Chang Y.-F., Ma S., Dembo A.G., Raj G.V. et al. Genomic agonism and phenotypic antagonism between estrogen and progesterone receptors in breast cancer. Sci. Adv. 2016; 2:e1501924. 27386569 10.1126/sciadv.1501924 PMC4928895

[5] West D.C. , Pan D., Tonsing-Carter E.Y., Hernandez K.M., Pierce C.F., Styke S.C., Bowie K.R., Garcia T.I., Kocherginsky M., Conzen S.D. GR and ER coactivation alters the expression of differentiation genes and associates with improved ER+ breast cancer outcome. Mol. Cancer Res. 2016; 14:707–719. 27141101 10.1158/1541-7786.MCR-15-0433 PMC5008962

[6] Peters A.A. , Buchanan G., Ricciardelli C., Bianco-Miotto T., Centenera M.M., Harris J.M., Jindal S., Segara D., Jia L., Moore N.L. et al. Androgen receptor inhibits estrogen Receptor-α activity and is prognostic in breast cancer. Cancer Res. 2009; 69:6131–6140. 19638585 10.1158/0008-5472.CAN-09-0452

[7] D’Amato N.C. , Gordon M.A., Babbs B., Spoelstra N.S., Butterfield K.T.C., Torkko K.C., Phan V.T., Barton V.N., Rogers T.J., Sartorius C.A. et al. Cooperative dynamics of AR and ER activity in breast cancer. Mol. Cancer Res. 2016; 14:1054–1067. 27565181 10.1158/1541-7786.MCR-16-0167 PMC5107172

[8] Hickey T.E. , Selth L.A., Chia K.M., Laven-Law G., Milioli H.H., Roden D., Jindal S., Hui M., Finlay-Schultz J., Ebrahimie E. et al. The androgen receptor is a tumor suppressor in estrogen receptor–positive breast cancer. Nat. Med. 2021; 27:310–320. 33462444 10.1038/s41591-020-01168-7

[9] Chung W. , Eum H.H., Lee H.-O., Lee K.-M., Lee H.-B., Kim K.-T., Ryu H.S., Kim S., Lee J.E., Park Y.H. et al. Single-cell RNA-seq enables comprehensive tumour and immune cell profiling in primary breast cancer. Nat. Commun. 2017; 8:15081. 28474673 10.1038/ncomms15081 PMC5424158

[10] Nguyen Q.H. , Pervolarakis N., Blake K., Ma D., Davis R.T., James N., Phung A.T., Willey E., Kumar R., Jabart E. et al. Profiling human breast epithelial cells using single cell RNA sequencing identifies cell diversity. Nat. Commun. 2018; 9:2028. 29795293 10.1038/s41467-018-04334-1 PMC5966421

[11] Casasent A.K. , Schalck A., Gao R., Sei E., Long A., Pangburn W., Casasent T., Meric-Bernstam F., Edgerton M.E., Navin N.E. Multiclonal invasion in breast tumors identified by topographic single cell sequencing. Cell. 2018; 172:205–217. 29307488 10.1016/j.cell.2017.12.007 PMC5766405

[12] Pal B. , Chen Y., Vaillant F., Capaldo B.D., Joyce R., Song X., Bryant V.L., Penington J.S., Di Stefano L., Tubau Ribera N. A single-cell RNA expression atlas of normal, preneoplastic and tumorigenic states in the human breast. EMBO J. 2021; 40:e107333. 33950524 10.15252/embj.2020107333 PMC8167363

[13] Wu S.Z. , Al-Eryani G., Roden D.L., Junankar S., Harvey K., Andersson A., Thennavan A., Wang C., Torpy J.R., Bartonicek N. et al. A single-cell and spatially resolved atlas of human breast cancers. Nat. Genet. 2021; 53:1334–1347. 34493872 10.1038/s41588-021-00911-1 PMC9044823

[14] Kim C. , Gao R., Sei E., Brandt R., Hartman J., Hatschek T., Crosetto N., Foukakis T., Navin N.E. Chemoresistance evolution in triple-negative breast cancer delineated by single-cell sequencing. Cell. 2018; 173:879–893. 29681456 10.1016/j.cell.2018.03.041 PMC6132060

[15] Giraddi R.R. , Chung C.-Y., Heinz R.E., Balcioglu O., Novotny M., Trejo C.L., Dravis C., Hagos B.M., Mehrabad E.M., Rodewald L.W. et al. Single-Cell transcriptomes distinguish stem cell state changes and lineage specification programs in early mammary gland development. Cell Rep. 2018; 24:1653–1666. 30089273 10.1016/j.celrep.2018.07.025 PMC6301014

[16] Yeo S.K. , Zhu X., Okamoto T., Hao M., Wang C., Lu P., Lu L.J., Guan J.-L. Single-cell RNA-sequencing reveals distinct patterns of cell state heterogeneity in mouse models of breast cancer. Elife. 2020; 9:e58810. 32840210 10.7554/eLife.58810 PMC7447441

[17] Zheng G.X.Y. , Terry J.M., Belgrader P., Ryvkin P., Bent Z.W., Wilson R., Ziraldo S.B., Wheeler T.D., McDermott G.P., Zhu J. et al. Massively parallel digital transcriptional profiling of single cells. Nat. Commun. 2017; 8:14049. 28091601 10.1038/ncomms14049 PMC5241818

[18] Trapnell C. , Cacchiarelli D., Grimsby J., Pokharel P., Li S., Morse M., Lennon N.J., Livak K.J., Mikkelsen T.S., Rinn J.L. The dynamics and regulators of cell fate decisions are revealed by pseudotemporal ordering of single cells. Nat. Biotechnol. 2014; 32:381–386. 24658644 10.1038/nbt.2859 PMC4122333

[19] Manno G.L. , Soldatov R., Zeisel A., Braun E., Hochgerner H., Petukhov V., Lidschreiber K., Kastriti M.E., Lönnerberg P., Furlan A. et al. RNA velocity of single cells. Nature. 2018; 560:494–498. 30089906 10.1038/s41586-018-0414-6 PMC6130801

[20] Street K. , Risso D., Fletcher R.B., Das D., Ngai J., Yosef N., Purdom E., Dudoit S. Slingshot: cell lineage and pseudotime inference for single-cell transcriptomics. BMC Genomics. 2018; 19:477. 29914354 10.1186/s12864-018-4772-0 PMC6007078

[21] Rulands S. , Lee H.J., Clark S.J., Angermueller C., Smallwood S.A., Krueger F., Mohammed H., Dean W., Nichols J., Rugg-Gunn P. et al. Genome-Scale oscillations in DNA methylation during exit from pluripotency. Cell Syst. 2018; 7:63–76. 30031774 10.1016/j.cels.2018.06.012 PMC6066359

[22] Guillen K.P. , Fujita M., Butterfield A.J., Scherer S.D., Bailey M.H., Chu Z., DeRose Y.S., Zhao L., Cortes-Sanchez E., Yang C.-H. et al. A human breast cancer-derived xenograft and organoid platform for drug discovery and precision oncology. Nat. Cancer. 2022; 3:232–250. 35221336 10.1038/s43018-022-00337-6 PMC8882468

[23] Satija R. , Farrell J.A., Gennert D., Schier A.F., Regev A. Spatial reconstruction of single-cell gene expression data. Nat. Biotechnol. 2015; 33:495–502. 25867923 10.1038/nbt.3192 PMC4430369

[24] Butler A. , Hoffman P., Smibert P., Papalexi E., Satija R. Integrating single-cell transcriptomic data across different conditions, technologies, and species. Nat. Biotechnol. 2018; 36:411–420. 29608179 10.1038/nbt.4096 PMC6700744

[25] Stuart T. , Butler A., Hoffman P., Hafemeister C., Papalexi E., Mauck W.M., Hao Y., Stoeckius M., Smibert P., Satija R. Comprehensive integration of single-cell data. Cell. 2019; 177:1888–1902. 31178118 10.1016/j.cell.2019.05.031 PMC6687398

[26] González-Blas C.B. , Minnoye L., Papasokrati D., Aibar S., Hulselmans G., Christiaens V., Davie K., Wouters J., Aerts S. cisTopic: cis-regulatory topic modeling on single-cell ATAC-seq data. Nat. Methods. 2019; 16:397–400. 30962623 10.1038/s41592-019-0367-1 PMC6517279

[27] Zhao W. , Chen J.J., Perkins R., Liu Z., Ge W., Ding Y., Zou W. A heuristic approach to determine an appropriate number of topics in topic modeling. BMC Bioinf. 2015; 16:S8–S8. 10.1186/1471-2105-16-S13-S8 PMC4597325 26424364

[28] Griffiths T.L. , Steyvers M. Finding scientific topics. Proc. Natl. Acad. Sci. U.S.A. 2004; 101:5228–5235. 14872004 10.1073/pnas.0307752101 PMC387300

[29] Mei S. , Qin Q., Wu Q., Sun H., Zheng R., Zang C., Zhu M., Wu J., Shi X., Taing L. et al. Cistrome data browser: a data portal for chip-Seq and chromatin accessibility data in human and mouse. Nucleic Acids Res. 2017; 45:D658–D662. 27789702 10.1093/nar/gkw983 PMC5210658

[30] Zheng R. , Wan C., Mei S., Qin Q., Wu Q., Sun H., Chen C.-H., Brown M., Zhang X., Meyer C.A. et al. Cistrome data browser: expanded datasets and new tools for gene regulatory analysis. Nucleic Acids Res. 2018; 47:D729–D735. 10.1093/nar/gky1094 PMC6324081 30462313

[31] Liu T. , Ortiz J.A., Taing L., Meyer C.A., Lee B., Zhang Y., Shin H., Wong S.S., Ma J., Lei Y. et al. Cistrome: an integrative platform for transcriptional regulation studies. Genome Biol. 2011; 12:R83. 21859476 10.1186/gb-2011-12-8-r83 PMC3245621

[32] Tirosh I. , Izar B., Prakadan S.M., Wadsworth II,M.H., Treacy D., Trombetta J.J., Rotem A., Rodman C., Lian C., Murphy G. et al. Dissecting the multicellular ecosystem of metastatic melanoma by single-cell RNA-seq. Science. 2016; 352:189–196. 27124452 10.1126/science.aad0501 PMC4944528

[33] Aibar S. , González-Blas C.B., Moerman T., Huynh-Thu V.A., Imrichova H., Hulselmans G., Rambow F., Marine J.-C., Geurts P., Aerts J. et al. SCENIC: single-cell regulatory network inference and clustering. Nat. Methods. 2016; 14:1083–1086. 10.1038/nmeth.4463 PMC5937676 28991892

[34] Dey K.K. , Hsiao C.J., Stephens M. Visualizing the structure of RNA-seq expression data using grade of membership models. PLoS Genet. 2017; 13:e1006599. 28333934 10.1371/journal.pgen.1006599 PMC5363805

[35] Xie Z. , Bailey A., Kuleshov M.V., Clarke D.J.B., Evangelista J.E., Jenkins S.L., Lachmann A., Wojciechowicz M.L., Kropiwnicki E., Jagodnik K.M. et al. Gene set knowledge discovery with enrichr. Curr. Protoc. 2021; 1:e90. 33780170 10.1002/cpz1.90 PMC8152575

[36] Chen E.Y. , Tan C.M., Kou Y., Duan Q., Wang Z., Meirelles G.V., Clark N.R., Ma’ayan A. Enrichr: interactive and collaborative HTML5 gene list enrichment analysis tool. BMC Bioinf. 2013; 14:128. 10.1186/1471-2105-14-128 PMC3637064 23586463

[37] Kuleshov M.V. , Jones M.R., Rouillard A.D., Fernandez N.F., Duan Q., Wang Z., Koplev S., Jenkins S.L., Jagodnik K.M., Lachmann A. et al. Enrichr: a comprehensive gene set enrichment analysis web server 2016 update. Nucleic Acids Res. 2016; 44:W90–W97. 27141961 10.1093/nar/gkw377 PMC4987924

[38] Moerman T. , Santos S.A., González-Blas C.B., Simm J., Moreau Y., Aerts J., Aerts S. GRNBoost2 and arboreto: efficient and scalable inference of gene regulatory networks. Bioinform. Oxf. Engl. 2018; 35:2159–2161. 10.1093/bioinformatics/bty916 30445495

[39] Stuart T. , Srivastava A., Madad S., Lareau C.A., Satija R. Single-cell chromatin state analysis with signac. Nat. Methods. 2021; 18:1333–1341. 34725479 10.1038/s41592-021-01282-5 PMC9255697

[40] Zhang Y. , Liu T., Meyer C.A., Eeckhoute J., Johnson D.S., Bernstein B.E., Nusbaum C., Myers R.M., Brown M., Li W. et al. Model-based analysis of chip-Seq (MACS). Genome Biol. 2008; 9:R137. 18798982 10.1186/gb-2008-9-9-r137 PMC2592715

[41] Gu Z. , Eils R., Schlesner M. Complex heatmaps reveal patterns and correlations in multidimensional genomic data. Bioinformatics. 2016; 32:2847–2849. 27207943 10.1093/bioinformatics/btw313

[42] Schep A.N. , Wu B., Buenrostro J.D., Greenleaf W.J. chromVAR: inferring transcription-factor-associated accessibility from single-cell epigenomic data. Nat. Methods. 2017; 14:975–978. 28825706 10.1038/nmeth.4401 PMC5623146

[43] Khan A. , Fornes O., Stigliani A., Gheorghe M., Castro-Mondragon J.A., Lee R.vander, Bessy A., Chèneby J., Kulkarni S.R., Tan G. et al. JASPAR 2018: update of the open-access database of transcription factor binding profiles and its web framework. Nucleic Acids Res. 2018; 46:D260–D266. 29140473 10.1093/nar/gkx1126 PMC5753243

[44] Pliner H.A. , Packer J.S., McFaline-Figueroa J.L., Cusanovich D.A., Daza R.M., Aghamirzaie D., Srivatsan S., Qiu X., Jackson D., Minkina A. et al. Cicero predicts cis-Regulatory DNA interactions from single-cell chromatin accessibility data. Mol. Cell. 2018; 71:858–871. 30078726 10.1016/j.molcel.2018.06.044 PMC6582963

[45] Duren Z. , Chang F., Naqing F., Xin J., Liu Q., Wong W.H. Regulatory analysis of single cell multiome gene expression and chromatin accessibility data with scREG. Genome Biol. 2022; 23:114. 35578363 10.1186/s13059-022-02682-2 PMC9109353

[46] Liu Q. , Chen S., Jiang R., Wong W.H. Simultaneous deep generative modelling and clustering of single-cell genomic data. Nat. Mach. Intell. 2021; 3:536–544. 34179690 10.1038/s42256-021-00333-y PMC8223760

[47] Lance C. , Luecken M.D., Burkhardt D.B., Cannoodt R., Rautenstrauch P., Laddach A., Ubingazhibov A., Cao Z.-J., Deng K., Khan S. et al. Multimodal single cell data integration challenge: results and lessons learned. Proceedings of the NeurIPS 2021 Competitions and Demonstrations Track. 2022; 176:Proceedings of Machine Learning Research 162–176.

[48] DeRose Y.S. , Wang G., Lin Y.-C., Bernard P.S., Buys S.S., Ebbert M.T.W., Factor R., Matsen C., Milash B.A., Nelson E. et al. Tumor grafts derived from women with breast cancer authentically reflect tumor pathology, growth, metastasis and disease outcomes. Nat. Med. 2011; 17:1514–1520. 22019887 10.1038/nm.2454 PMC3553601

[49] Becht E. , McInnes L., Healy J., Dutertre C.-A., Kwok I.W.H., Ng L.G., Ginhoux F., Newell E.W. Dimensionality reduction for visualizing single-cell data using UMAP. Nat. Biotechnol. 2018; 37:38–44. 10.1038/nbt.4314 30531897

[50] Hurtado A. , Holmes K.A., Ross-Innes C.S., Schmidt D., Carroll J.S. FOXA1 is a key determinant of estrogen receptor function and endocrine response. Nat. Genet. 2011; 43:27–33. 21151129 10.1038/ng.730 PMC3024537

[51] Stope M.B. , Popp S.L., Knabbe C., Buck M.B. Estrogen receptor alpha attenuates transforming growth factor-beta signaling in breast cancer cells independent from agonistic and antagonistic ligands. Breast Cancer Res. Tr. 2008; 120:357–367. 10.1007/s10549-009-0393-2 19370415

[52] Wang C. , Mayer J.A., Mazumdar A., Fertuck K., Kim H., Brown M., Brown P.H. Estrogen induces c-myc gene expression via an upstream enhancer activated by the estrogen receptor and the AP-1 transcription factor. Mol. Endocrinol. 2011; 25:1527–1538. 21835891 10.1210/me.2011-1037 PMC3165912

[53] Sanders D.A. , Ross-Innes C.S., Beraldi D., Carroll J.S., Balasubramanian S. Genome-wide mapping of FOXM1 binding reveals co-binding with estrogen receptor alpha in breast cancer cells. Genome Biol. 2013; 14:R6. 23347430 10.1186/gb-2013-14-1-r6 PMC3663086

[54] Madureira P.A. , Varshochi R., Constantinidou D., Francis R.E., Coombes R.C., Yao K.-M., Lam E.W.-F. The forkhead box M1 protein regulates the transcription of the estrogen receptor α in breast cancer cells. J. Biol. Chem. 2006; 281:25167–25176. 16809346 10.1074/jbc.M603906200

[55] Cordera F. , Jordan V.C. Steroid receptors and their role in the biology and control of breast cancer growth. Semin. Oncol. 2006; 33:631–641. 17145341 10.1053/j.seminoncol.2006.08.020

[56] Smith S.E. , Mellor P., Ward A.K., Kendall S., McDonald M., Vizeacoumar F.S., Vizeacoumar F.J., Napper S., Anderson D.H. Molecular characterization of breast cancer cell lines through multiple omic approaches. Breast Cancer Res. 2017; 19:65. 28583138 10.1186/s13058-017-0855-0 PMC5460504

[57] Saldivar J.C. , Hamperl S., Bocek M.J., Chung M., Bass T.E., Cisneros-Soberanis F., Samejima K., Xie L., Paulson J.R., Earnshaw W.C. et al. An intrinsic S/G2 checkpoint enforced by ATR. Science. 2018; 361:806–810. 30139873 10.1126/science.aap9346 PMC6365305

[58] Sullivan C. , Liu Y., Shen J., Curtis A., Newman C., Hock J.M., Li X. Novel interactions between FOXM1 and CDC25A regulate the cell cycle. PLoS One. 2012; 7:e51277. 23240008 10.1371/journal.pone.0051277 PMC3519786

[59] Stoica G.E. , Franke T.F., Moroni M., Mueller S., Morgan E., Iann M.C., Winder A.D., Reiter R., Wellstein A., Martin M.B. et al. Effect of estradiol on estrogen receptor-alpha gene expression and activity can be modulated by the ErbB2/PI 3-K/Akt pathway. Oncogene. 2003; 22:7998–8011. 12970748 10.1038/sj.onc.1206769

[60] Mori H. , Saeki K., Chang G., Wang J., Wu X., Hsu P.-Y., Kanaya N., Wang X., Somlo G., Nakamura M. et al. Influence of estrogen treatment on ESR1+ and ESR1− cells in ER+ breast cancer: insights from single-cell analysis of patient-derived xenograft models. Cancers. 2021; 13:6375. 34944995 10.3390/cancers13246375 PMC8699443

[61] Ahn H. , Sim J., Abdul R., Chung M.S., Paik S.S., Oh Y.-H., Park C.K., Jang K. Increased expression of forkhead box M1 is associated with aggressive phenotype and poor prognosis in estrogen receptor-positive breast cancer. J. Korean Med. Sci. 2014; 30:390–397. 10.3346/jkms.2015.30.4.390 PMC4366959 25829806

[62] Ziegler Y. , Laws M.J., Guillen V.S., Kim S.H., Dey P., Smith B.P., Gong P., Bindman N., Zhao Y., Carlson K. et al. Suppression of FOXM1 activities and breast cancer growth in vitro and in vivo by a new class of compounds. Npj Breast Cancer. 2019; 5:45. 31815181 10.1038/s41523-019-0141-7 PMC6884575

[63] Sanders D.A. , Gormally M.V., Marsico G., Beraldi D., Tannahill D., Balasubramanian S. FOXM1 binds directly to non-consensus sequences in the human genome. Genome Biol. 2015; 16:130. 26100407 10.1186/s13059-015-0696-z PMC4492089

[64] Lei J.T. , Anurag M., Haricharan S., Gou X., Ellis M.J. Endocrine therapy resistance: new insights. Breast Edinb. Scotl. 2019; 48(Suppl. 1):S26–S30. 10.1016/S0960-9776(19)31118-X PMC6939855 31839155

[65] Anurag M. , Ellis M.J., Haricharan S. DNA damage repair defects as a new class of endocrine treatment resistance driver. Oncotarget. 2018; 9:36252–36253. 30555626 10.18632/oncotarget.26363 PMC6284739

[66] Lindström L.S. , Yau C., Czene K., Thompson C.K., Hoadley K.A., Veer L.J.V., Balassanian R., Bishop J.W., Carpenter P.M., Chen Y.-Y. et al. Intratumor heterogeneity of the estrogen receptor and the Long-term risk of fatal breast cancer. Jnci. J. Natl. Cancer Inst. 2018; 110:726–733. 29361175 10.1093/jnci/djx270 PMC6037086

[67] Chung G.G. , Zerkowski M.P., Ghosh S., Camp R.L., Rimm D.L. Quantitative analysis of estrogen receptor heterogeneity in breast cancer. Lab. Invest. 2007; 87:662–669. 17334408 10.1038/labinvest.3700543

[68] Millour J. , Constantinidou D., Stavropoulou A.V., Wilson M.S.C., Myatt S.S., Kwok J.M.-M., Sivanandan K., Coombes R.C., Medema R.H., Hartman J. et al. FOXM1 is a transcriptional target of ERalpha and has a critical role in breast cancer endocrine sensitivity and resistance. Oncogene. 2010; 29:2983–2995. 20208560 10.1038/onc.2010.47 PMC2874720

[69] Horimoto Y. , Hartman J., Millour J., Pollock S., Olmos Y., Ho K.-K., Coombes R.C., Poutanen M., Mäkelä S.I., El-Bahrawy M. et al. ERβ1 represses FOXM1 expression through targeting ERα to control cell proliferation in breast cancer. Am. J. Pathol. 2011; 179:1148–1156. 21763263 10.1016/j.ajpath.2011.05.052 PMC3157253

[70] Finlay-Schultz J. , Gillen A.E., Brechbuhl H.M., Ivie J.J., Matthews S.B., Jacobsen B.M., Bentley D.L., Kabos P., Sartorius C.A. Breast cancer suppression by progesterone receptors is mediated by their modulation of estrogen receptors and RNA polymerase III. Cancer Res. 2017; 77:4934–4946. 28729413 10.1158/0008-5472.CAN-16-3541 PMC5600857

[71] Lange C.A. , Yee D. Progesterone and breast cancer. Women's Heal. 2008; 4:151–162. 10.2217/17455057.4.2.151 PMC4038907 19072517

[72] Narayanan R. , Edwards D.P., Weigel N.L. Human progesterone receptor displays cell cycle-dependent changes in transcriptional activity. Mol. Cell. Biol. 2005; 25:2885–2898. 15798179 10.1128/MCB.25.8.2885-2898.2005 PMC1069605

[73] Ogara M.F. , Rodríguez-Seguí S.A., Marini M., Nacht A.S., Stortz M., Levi V., Presman D.M., Vicent G.P., Pecci A. The glucocorticoid receptor interferes with progesterone receptor-dependent genomic regulation in breast cancer cells. Nucleic Acids Res. 2019; 47:10645–10661. 31598691 10.1093/nar/gkz857 PMC6846950

[74] Hammond M.E.H. , Hayes D.F., Dowsett M., Allred D.C., Hagerty K.L., Badve S., Fitzgibbons P.L., Francis G., Goldstein N.S., Hayes M. et al. American society of clinical oncology/college of american pathologists guideline recommendations for immunohistochemical testing of estrogen and progesterone receptors in breast cancer (Unabridged version). Arch. Pathol. Lab. Med. 2010; 134:e48–e72. 20586616 10.5858/134.7.e48

[75] Harvey J.M. , Clark G.M., Osborne C.K., Allred D.C. Estrogen receptor status by immunohistochemistry is superior to the ligand-binding assay for predicting response to adjuvant endocrine therapy in breast cancer. J. Clin. Oncol. Official. J. Am. Soc. Clin. Oncol. 1999; 17:1474–1481. 10.1200/JCO.1999.17.5.1474 10334533

[76] Yi M. , Huo L., Koenig K.B., Mittendorf E.A., Meric-Bernstam F., Kuerer H.M., Bedrosian I., Buzdar A.U., Symmans W.F., Crow J.R. et al. Which threshold for ER positivity? a retrospective study based on 9639 patients. Ann. Oncol. 2014; 25:1004–1011. 24562447 10.1093/annonc/mdu053 PMC3999801

[77] Raghav K.P.S. , Hernandez-Aya L.F., Lei X., Chavez-MacGregor M., Meric-Bernstam F., Buchholz T.A., Sahin A., Do K., Hortobagyi G.N., Gonzalez-Angulo A.M. Impact of low estrogen/progesterone receptor expression on survival outcomes in breast cancers previously classified as triple negative breast cancers. Cancer. 2012; 118:1498–1506. 21837669 10.1002/cncr.26431 PMC3217101

[78] Vantaggiato C. , Tocchetti M., Cappelletti V., Gurtner A., Villa A., Daidone M.G., Piaggio G., Maggi A., Ciana P. Cell cycle dependent oscillatory expression of estrogen receptor-α links pol II elongation to neoplastic transformation. Proc. Natl. Acad. Sci. U.S.A. 2014; 111:9561–9566. 24979764 10.1073/pnas.1321750111 PMC4084491

