# Supplementary material for: Estrogen regulates divergent transcriptional and epigenetic cell states in breast cancer
Source: Nucleic Acids Res. 2022 Nov 1;50(20):11492–508. doi: 10.1093/nar/gkac908 (PMC9723652; doi:10.1093/nar/gkac908)
Supplement: gkac908_Supplemental_Files [file gkac908_supplemental_files.zip › SuppFigures_1-10_legends.pdf]

# S1

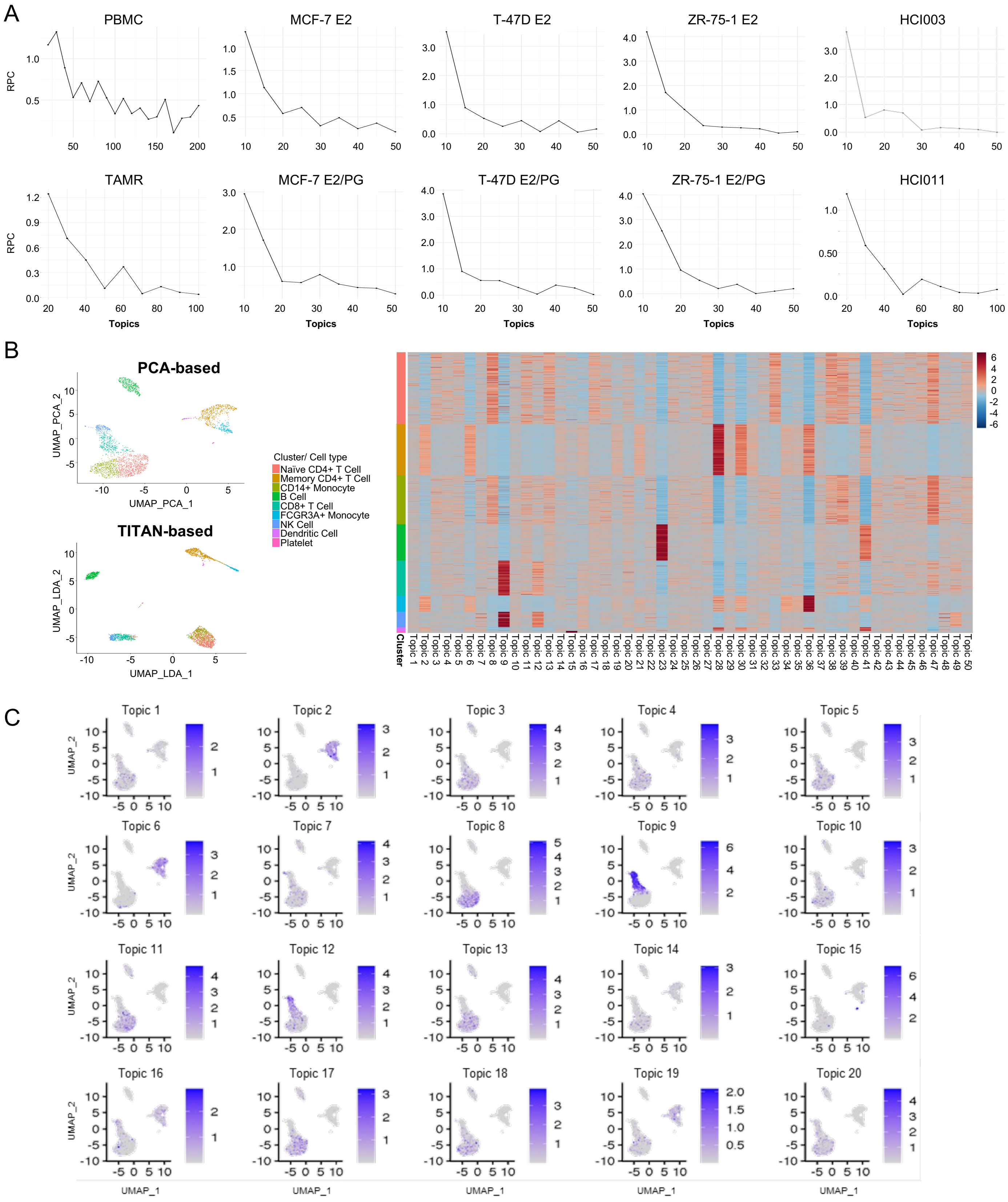
A
PBMC
MCF-7 E2
T-47D E2
ZR-75-1 E2
HCI003
TAMR
MCF-7 E2/PG
T-47D E2/PG
ZR-75-1 E2/PG
HCI011
RPC
Topics
B
PCA-based
TITAN-based
UMAP_PCA_1
UMAP_PCA_2
UMAP_LDA_1
UMAP_LDA_2
Cluster/ Cell type
Naïve CD4+ T Cell
Memory CD4+ T Cell
CD14+ Monocyte
B Cell
CD8+ T Cell
FCGR3A+ Monocyte
NK Cell
Dendritic Cell
Platelet
Cluster
Topic 1 … Topic 50
C
Topic 1
Topic 2
Topic 3
Topic 4
Topic 5
Topic 6
Topic 7
Topic 8
Topic 9
Topic 10
Topic 11
Topic 12
Topic 13
Topic 14
Topic 15
Topic 16
Topic 17
Topic 18
Topic 19
Topic 20
UMAP_1
UMAP_2

**S2**

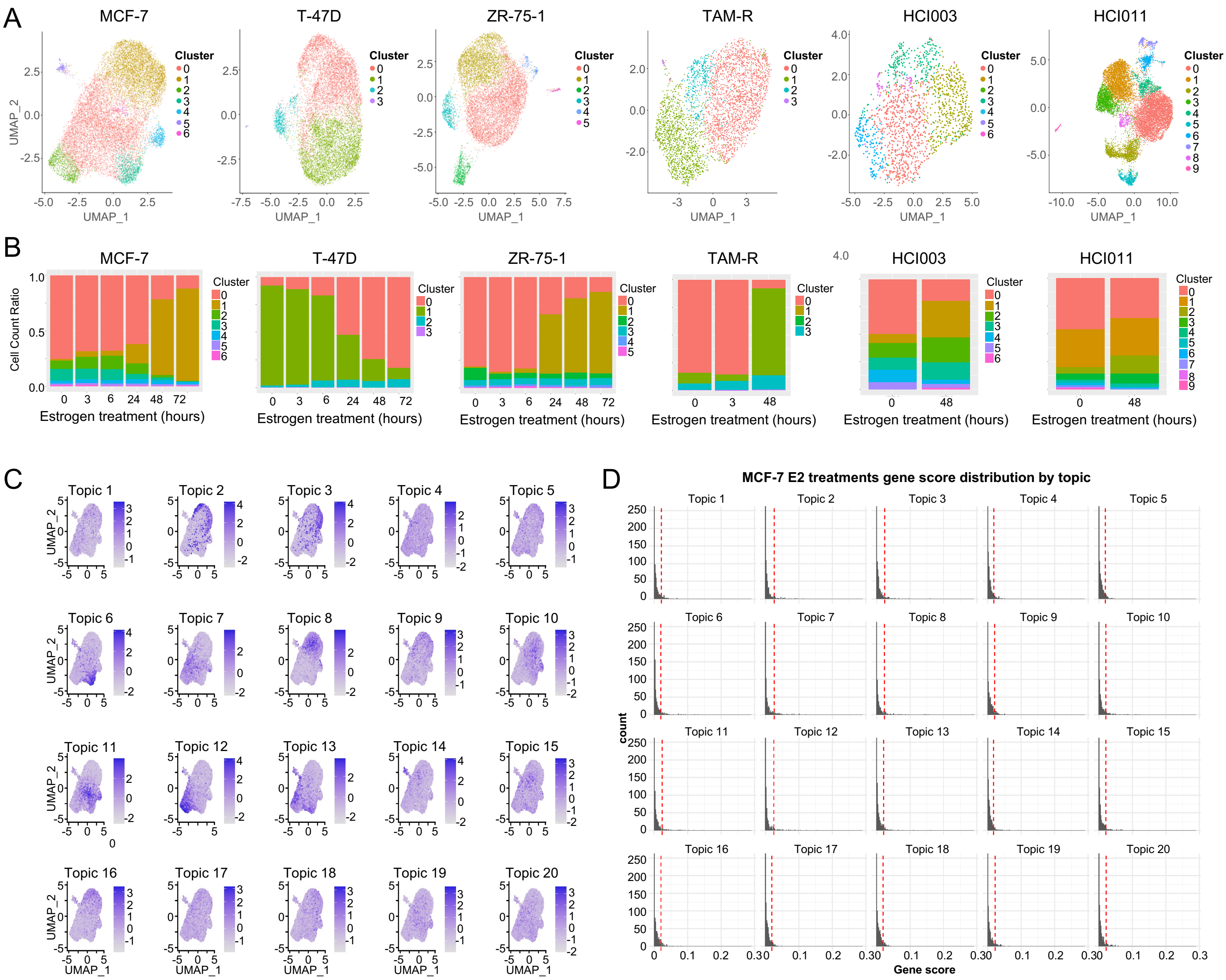


E

**Topic 8 – Upregulated by estrogen**

| Term | P-value |
|---|---|
| Validated nuclear estrogen receptor alpha network | 1.2E-08 |
| HNF3A pathway | 1.8E-04 |
| Sphingolipid metabolism | 7.8E-04 |
| Downregulation of MTA-3 in ER-negative breast tumors | 9.1E-04 |
| Estrogen receptor transcription factor targets | 1.9E-03 |
| Ovarian infertility genes | 2.5E-03 |
| Glycosphingolipid metabolism | 4.1E-03 |
| Trefoil factor initiation of mucosal healing | 4.1E-03 |
| Interleukin-2 signaling pathway | 4.9E-03 |

**Topic 13 – Downregulated by estrogen**

| Term | P-value |
|---|---|
| Myc repressed pathway | 1.05E-06 |
| BDNF signaling pathway | 3.68E-06 |
| TSH regulation of gene expression | 4.31E-06 |
| Signaling events mediated by PRL | 2.51E-05 |
| EGFR1 pathway | 3.82E-05 |
| Gastrin pathway | 1.81E-04 |
| Prolactin regulation of apoptosis | 2.15E-04 |
| TGF-beta regulation of extracellular matrix | 4.82E-04 |
| Signaling by TGF-beta receptor complex | 6.02E-04 |

S3

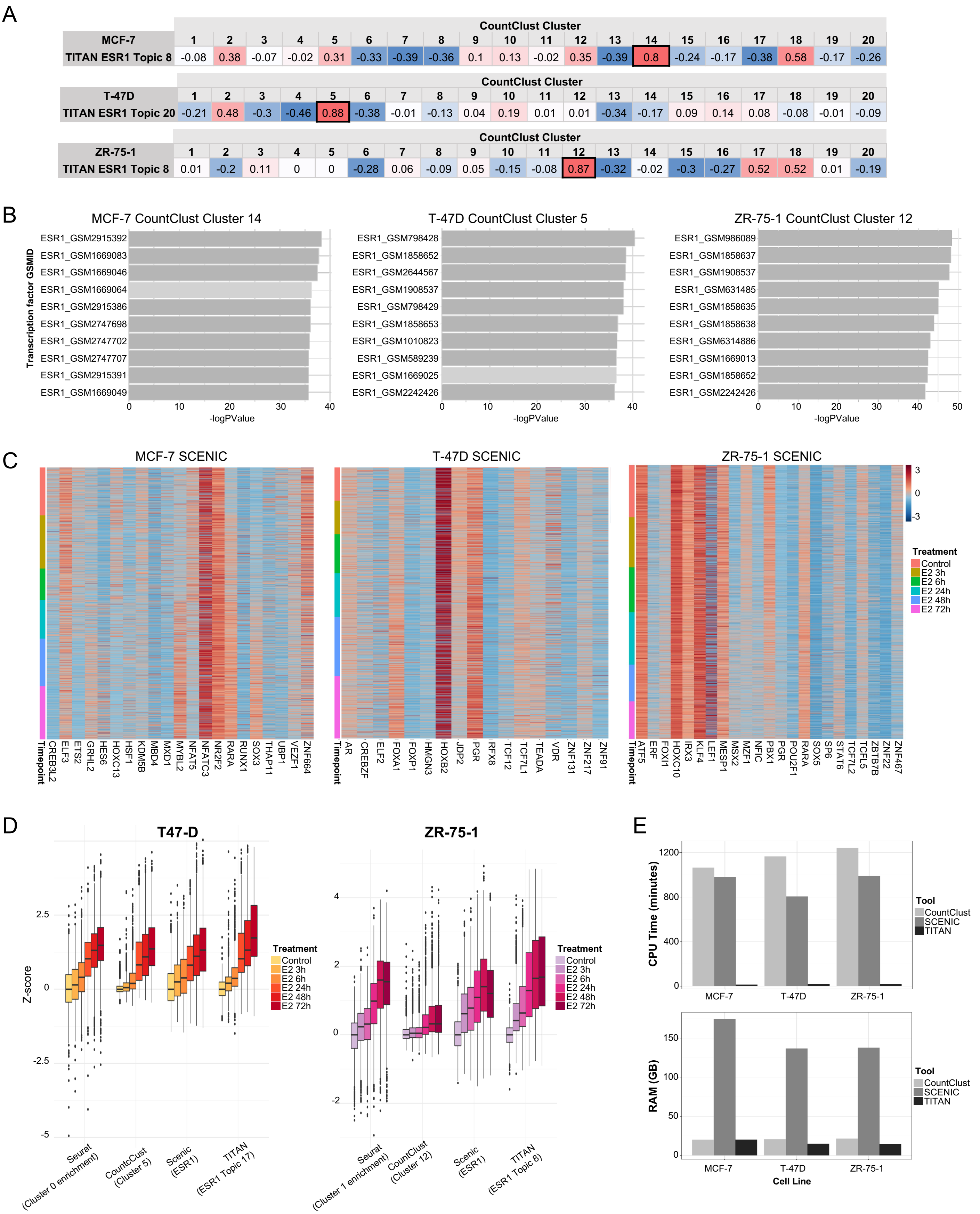

A
CountClust Cluster
MCF-7
1 2 3 4 5 6 7 8 9 10 11 12 13 14 15 16 17 18 19 20
TITAN ESR1 Topic 8 -0.08 0.38 -0.07 -0.02 0.31 -0.33 -0.39 -0.36 0.1 0.13 -0.02 0.35 -0.39 0.8 -0.24 -0.17 -0.38 0.58 -0.17 -0.26
CountClust Cluster
T-47D
1 2 3 4 5 6 7 8 9 10 11 12 13 14 15 16 17 18 19 20
TITAN ESR1 Topic 20 -0.21 0.48 -0.3 -0.46 0.88 -0.38 -0.01 -0.13 0.04 0.19 0.01 0.01 -0.34 -0.17 0.09 0.14 0.08 -0.08 -0.01 -0.09
CountClust Cluster
ZR-75-1
1 2 3 4 5 6 7 8 9 10 11 12 13 14 15 16 17 18 19 20
TITAN ESR1 Topic 8 0.01 -0.2 0.11 0 0 -0.28 0.06 -0.09 0.05 -0.15 -0.08 0.87 -0.32 -0.02 -0.3 -0.27 0.52 0.52 0.01 -0.19
B
MCF-7 CountClust Cluster 14
Transcription factor GSMID
ESR1_GSM2915392
ESR1_GSM1669083
ESR1_GSM1669046
ESR1_GSM1669064
ESR1_GSM2915386
ESR1_GSM2747698
ESR1_GSM2747702
ESR1_GSM2747707
ESR1_GSM2915391
ESR1_GSM1669049
0 10 20 30 40
-logPValue
T-47D CountClust Cluster 5
ESR1_GSM798428
ESR1_GSM1858652
ESR1_GSM2644567
ESR1_GSM1908537
ESR1_GSM798429
ESR1_GSM1858653
ESR1_GSM1010823
ESR1_GSM589239
ESR1_GSM1669025
ESR1_GSM2242426
0 10 20 30 40
-logPValue
ZR-75-1 CountClust Cluster 12
ESR1_GSM986089
ESR1_GSM1858637
ESR1_GSM1908537
ESR1_GSM631485
ESR1_GSM1858635
ESR1_GSM1858638
ESR1_GSM6314886
ESR1_GSM1669013
ESR1_GSM1858652
ESR1_GSM2242426
0 10 20 30 40 50
-logPValue
C
MCF-7 SCENIC
Timepoint CREB3L2 ELF3 ETS2 GRHL2 HES6 HOXC13 HSF1 KDM5B MBD4 MXD1 MYBL2 NFAT5 NFATC3 NR2F2 RARA RUNX1 SOX3 THAP11 UBP1 VEZF1 ZNF664
T-47D SCENIC
Timepoint AR CREBZF ELF2 FOXA1 FOXP1 HMGN3 HOXB2 JDP2 PGR RFX8 TCF12 TCF7L1 TEADA VDR ZNF131 ZNF217 ZNF91
ZR-75-1 SCENIC
Timepoint ATF5 ERF FOXI1 HOXC10 IRX3 KLF4 LEF1 MESP1 MSX2 MZF1 NFIC PBX1 PGR POU2F1 RARA SOX5 SP6 STAT6 TCF7L2 TCF7L5 ZBTB7B ZNF22 ZNF467
3
0
-3
Treatment
Control
E2 3h
E2 6h
E2 24h
E2 48h
E2 72h
D
T47-D
Z-score
2.5
0
-2.5
-5
Seurat (Cluster 0 enrichment)
CountCust (Cluster 5)
Scenic (ESR1)
TITAN (ESR1 Topic 17)
Treatment
Control
E2 3h
E2 6h
E2 24h
E2 48h
E2 72h
ZR-75-1
4
2
0
-2
Seurat (Cluster 1 enrichment)
CountClust (Cluster 12)
Scenic (ESR1)
TITAN (ESR1 Topic 8)
Treatment
Control
E2 3h
E2 6h
E2 24h
E2 48h
E2 72h
E
CPU Time (minutes)
1200
800
400
0
MCF-7 T-47D ZR-75-1
Tool
CountClust
SCENIC
TITAN
RAM (GB)
150
100
50
0
MCF-7 T-47D ZR-75-1
Cell Line
Tool
CountClust
SCENIC
TITAN

**S4**

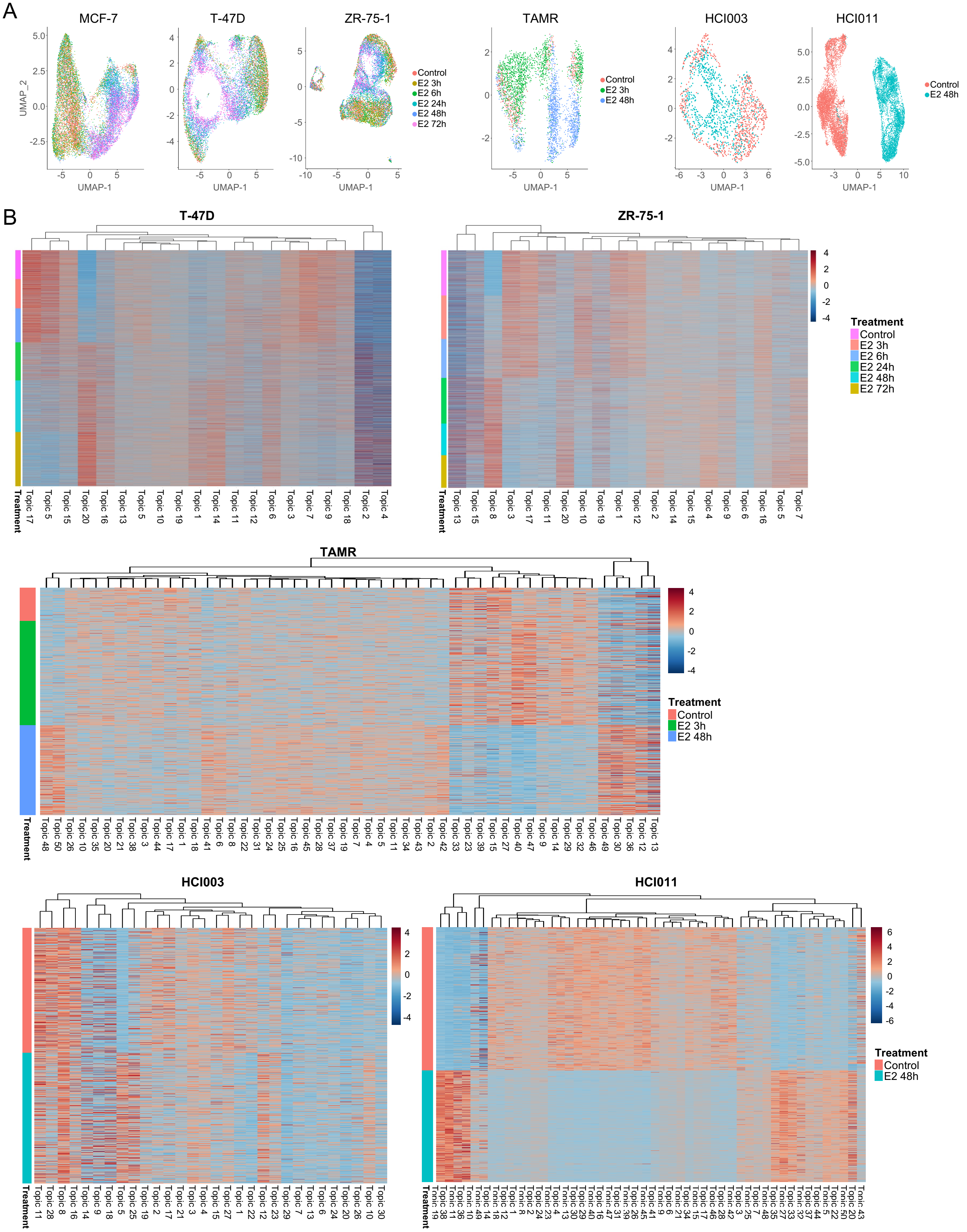
A
MCF-7
T-47D
ZR-75-1
TAMR
HCI003
HCI011
UMAP_2
UMAP-1
Control
E2 3h
E2 6h
E2 24h
E2 48h
E2 72h
B
T-47D
ZR-75-1
TAMR
HCI003
HCI011
Treatment
Control
E2 3h
E2 6h
E2 24h
E2 48h
E2 72h

# S5

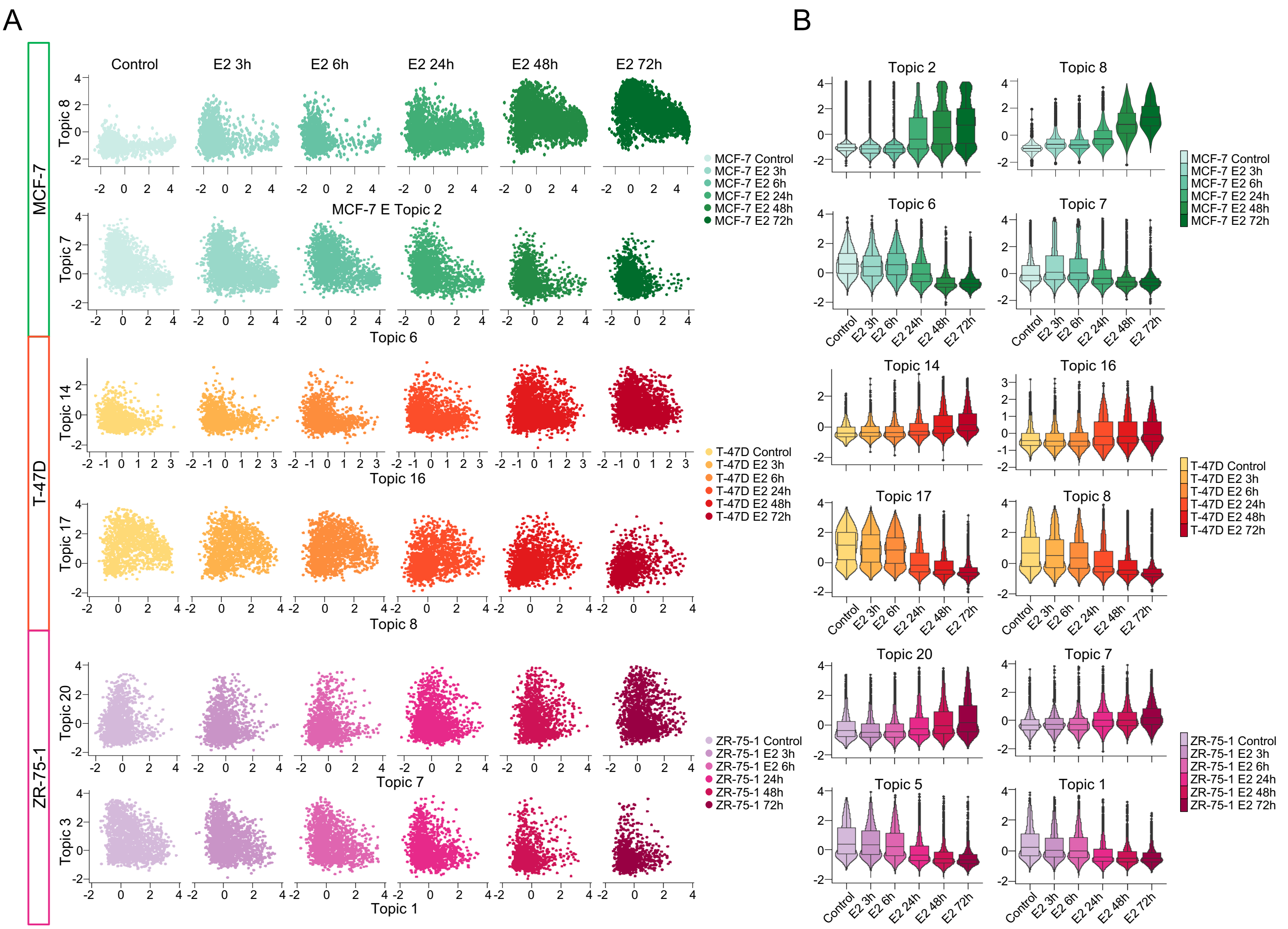
A
B
MCF-7
T-47D
ZR-75-1
Control
E2 3h
E2 6h
E2 24h
E2 48h
E2 72h
Topic 8
Topic 7
Topic 6
MCF-7 E Topic 2
Topic 14
Topic 16
Topic 17
Topic 8
Topic 20
Topic 7
Topic 3
Topic 1
MCF-7 Control
MCF-7 E2 3h
MCF-7 E2 6h
MCF-7 E2 24h
MCF-7 E2 48h
MCF-7 E2 72h
T-47D Control
T-47D E2 3h
T-47D E2 6h
T-47D E2 24h
T-47D E2 48h
T-47D E2 72h
ZR-75-1 Control
ZR-75-1 E2 3h
ZR-75-1 E2 6h
ZR-75-1 24h
ZR-75-1 48h
ZR-75-1 72h
Topic 2
Topic 8
Topic 6
Topic 7
Topic 14
Topic 16
Topic 17
Topic 8
Topic 20
Topic 7
Topic 5
Topic 1
ZR-75-1 E2 24h
ZR-75-1 E2 48h
ZR-75-1 E2 72h

**S6**

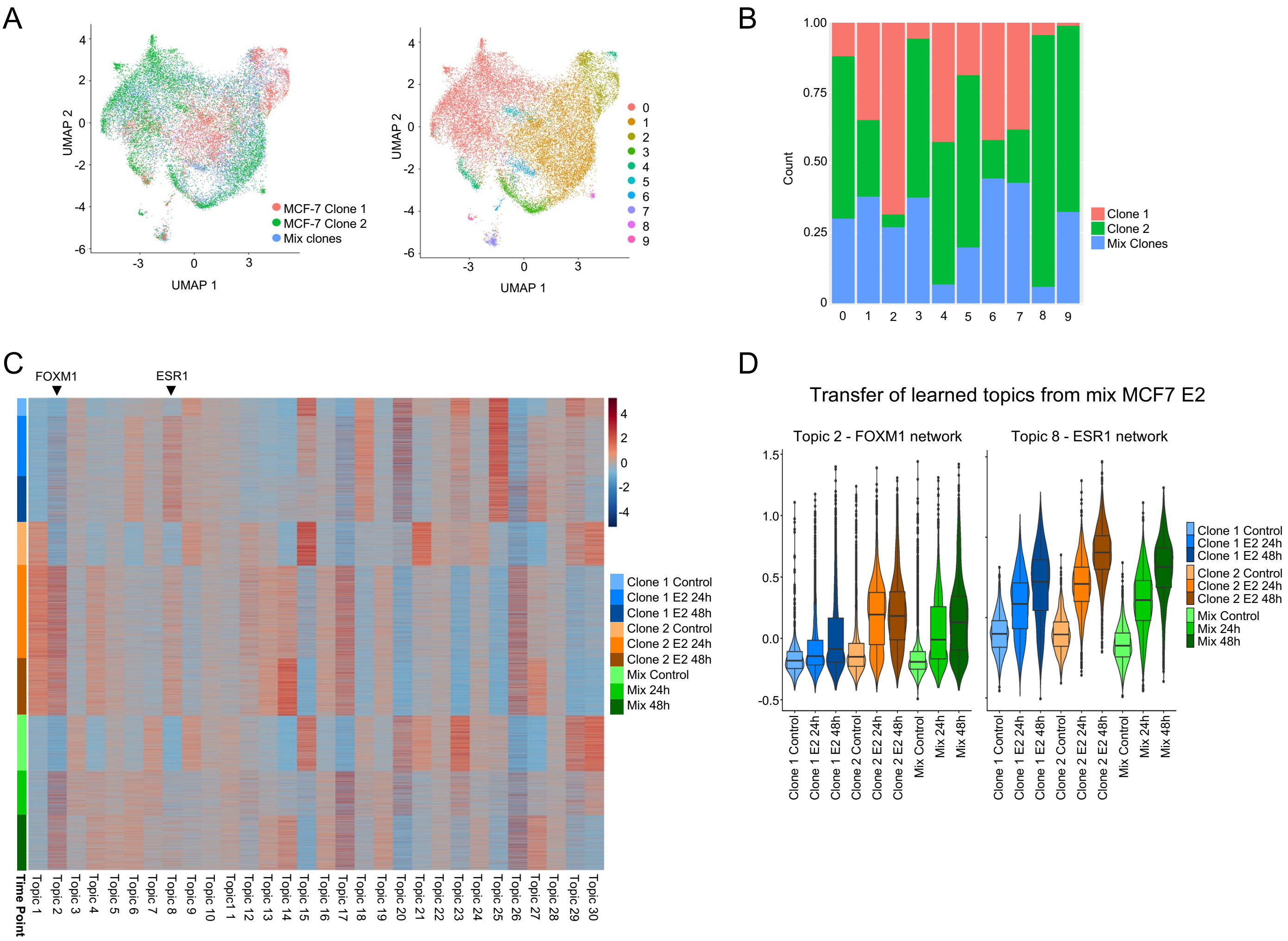

A
UMAP 1
UMAP 2
MCF-7 Clone 1
MCF-7 Clone 2
Mix clones
0 1 2 3 4 5 6 7 8 9
B
Count
1.00
0.75
0.50
0.25
0
Clone 1
Clone 2
Mix Clones
C
FOXM1
ESR1
Clone 1 Control
Clone 1 E2 24h
Clone 1 E2 48h
Clone 2 Control
Clone 2 E2 24h
Clone 2 E2 48h
Mix Control
Mix 24h
Mix 48h
Time Point
Topic 1 Topic 2 Topic 3 Topic 4 Topic 5 Topic 6 Topic 7 Topic 8 Topic 9 Topic 10 Topic1 1 Topic 12 Topic 13 Topic 14 Topic 15 Topic 16 Topic 17 Topic 18 Topic 19 Topic 20 Topic 21 Topic 22 Topic 23 Topic 24 Topic 25 Topic 26 Topic 27 Topic 28 Topic 29 Topic 30
D
Transfer of learned topics from mix MCF7 E2
Topic 2 - FOXM1 network
Topic 8 - ESR1 network
1.5
1.0
0.5
0.0
-0.5
Clone 1 Control
Clone 1 E2 24h
Clone 1 E2 48h
Clone 2 Control
Clone 2 E2 24h
Clone 2 E2 48h
Mix Control
Mix 24h
Mix 48h

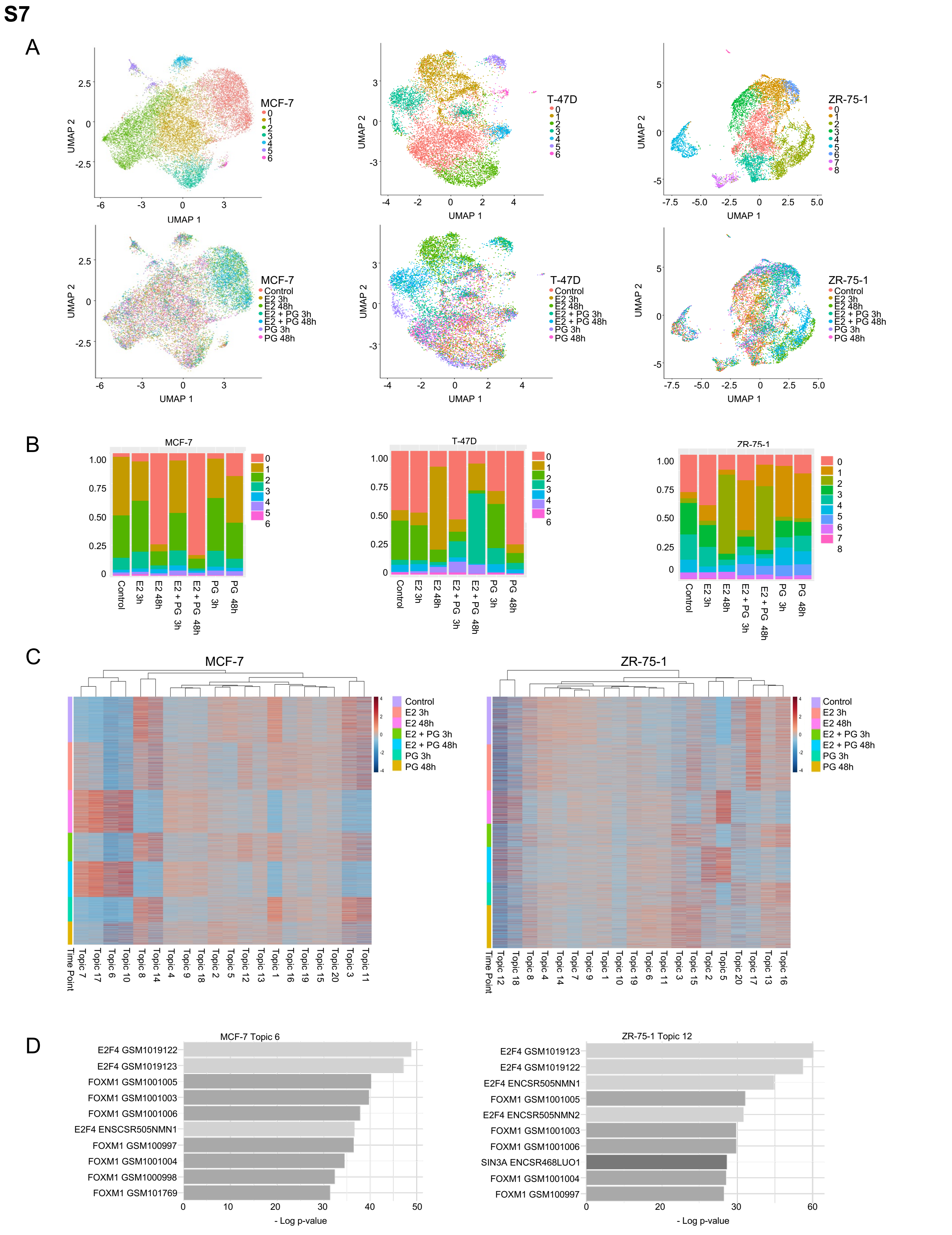
S7
A
MCF-7
T-47D
ZR-75-1
UMAP 1
UMAP 2
Control
E2 3h
E2 48h
E2 + PG 3h
E2 + PG 48h
PG 3h
PG 48h
B
MCF-7
T-47D
ZR-75-1
C
MCF-7
ZR-75-1
Time Point
D
MCF-7 Topic 6
E2F4 GSM1019122
E2F4 GSM1019123
FOXM1 GSM1001005
FOXM1 GSM1001003
FOXM1 GSM1001006
E2F4 ENSCSR505NMN1
FOXM1 GSM100997
FOXM1 GSM1001004
FOXM1 GSM1000998
FOXM1 GSM101769
- Log p-value
ZR-75-1 Topic 12
E2F4 GSM1019123
E2F4 GSM1019122
E2F4 ENCSR505NMN1
FOXM1 GSM1001005
E2F4 ENCSR505NMN2
FOXM1 GSM1001003
FOXM1 GSM1001006
SIN3A ENCSR468LUO1
FOXM1 GSM1001004
FOXM1 GSM100997
- Log p-value

**S8**

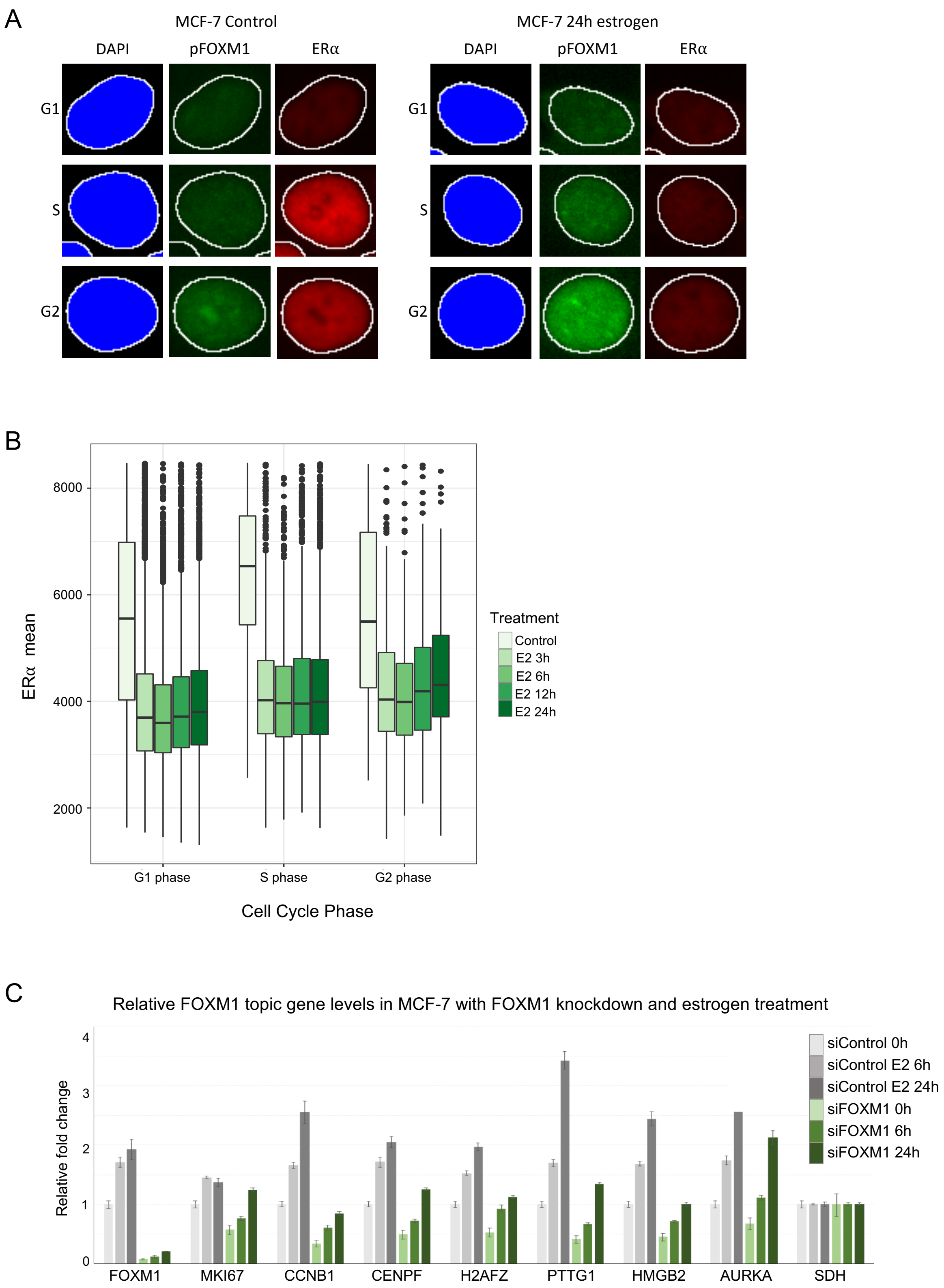

A
MCF-7 Control
MCF-7 24h estrogen
DAPI
pFOXM1
ERα
G1
S
G2
B
ERα mean
8000
6000
4000
2000
G1 phase
S phase
G2 phase
Cell Cycle Phase
Treatment
Control
E2 3h
E2 6h
E2 12h
E2 24h
C
Relative FOXM1 topic gene levels in MCF-7 with FOXM1 knockdown and estrogen treatment
Relative fold change
4
3
2
1
0
FOXM1
MKI67
CCNB1
CENPF
H2AFZ
PTTG1
HMGB2
AURKA
SDH
siControl 0h
siControl E2 6h
siControl E2 24h
siFOXM1 0h
siFOXM1 6h
siFOXM1 24h

**S9**

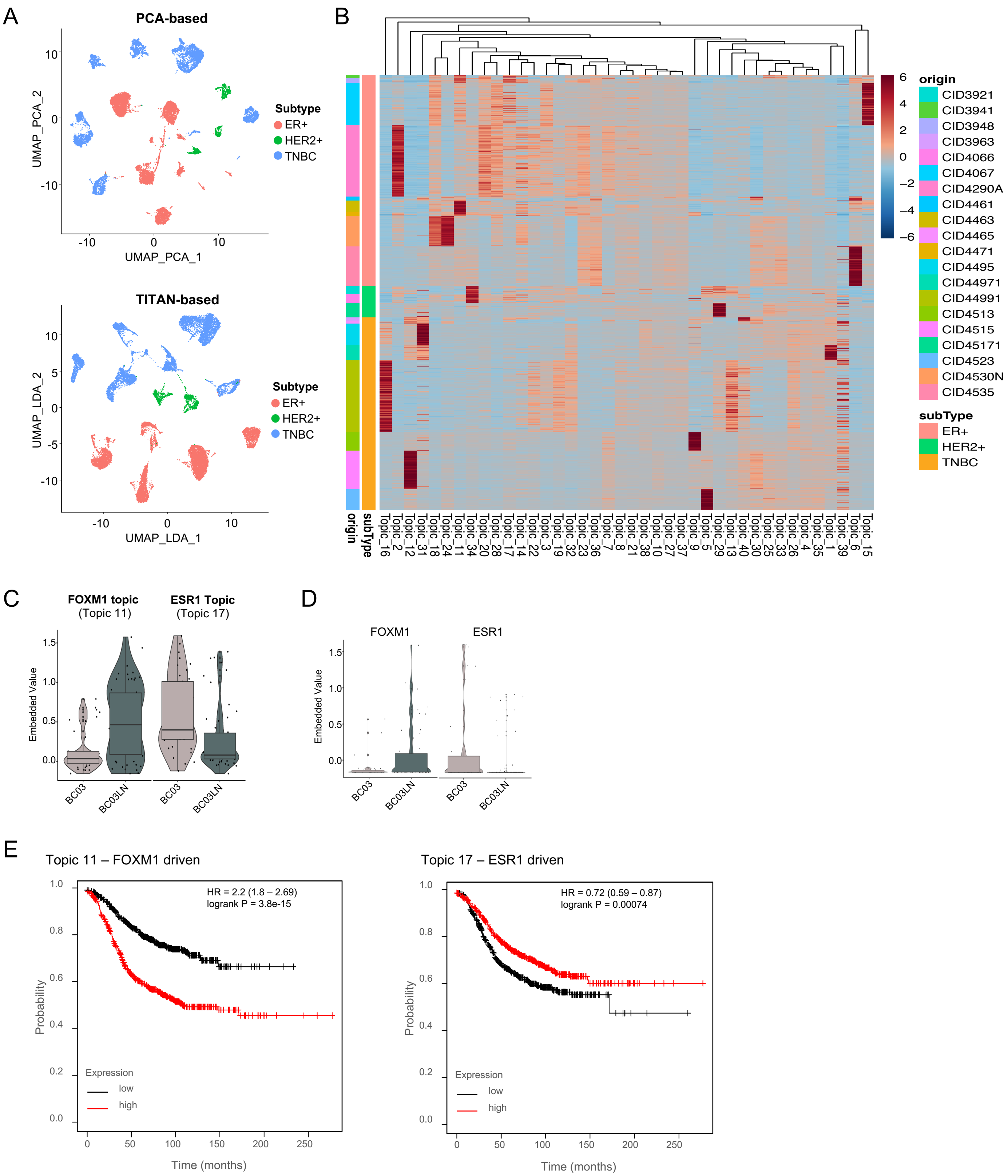
A
PCA-based
UMAP_PCA_1
UMAP_PCA_2
Subtype
ER+
HER2+
TNBC
TITAN-based
UMAP_LDA_1
UMAP_LDA_2
B
origin
CID3921
CID3941
CID3948
CID3963
CID4066
CID4067
CID4290A
CID4461
CID4463
CID4465
CID4471
CID4495
CID44971
CID44991
CID4513
CID4515
CID45171
CID4523
CID4530N
CID4535
subType
ER+
HER2+
TNBC
Topic_16 Topic_2 Topic_12 Topic_31 Topic_18 Topic_24 Topic_11 Topic_34 Topic_20 Topic_28 Topic_17 Topic_14 Topic_22 Topic_3 Topic_19 Topic_32 Topic_23 Topic_36 Topic_7 Topic_8 Topic_21 Topic_38 Topic_10 Topic_27 Topic_37 Topic_9 Topic_5 Topic_29 Topic_13 Topic_40 Topic_30 Topic_25 Topic_33 Topic_26 Topic_4 Topic_35 Topic_1 Topic_39 Topic_6 Topic_15
C
FOXM1 topic
(Topic 11)
ESR1 Topic
(Topic 17)
Embedded Value
BC03
BC03LN
D
FOXM1
ESR1
Embedded Value
BC03
BC03LN
E
Topic 11 – FOXM1 driven
HR = 2.2 (1.8 – 2.69)
logrank P = 3.8e-15
Probability
Time (months)
Expression
low
high
Topic 17 – ESR1 driven
HR = 0.72 (0.59 – 0.87)
logrank P = 0.00074
Probability
Time (months)
Expression
low
high

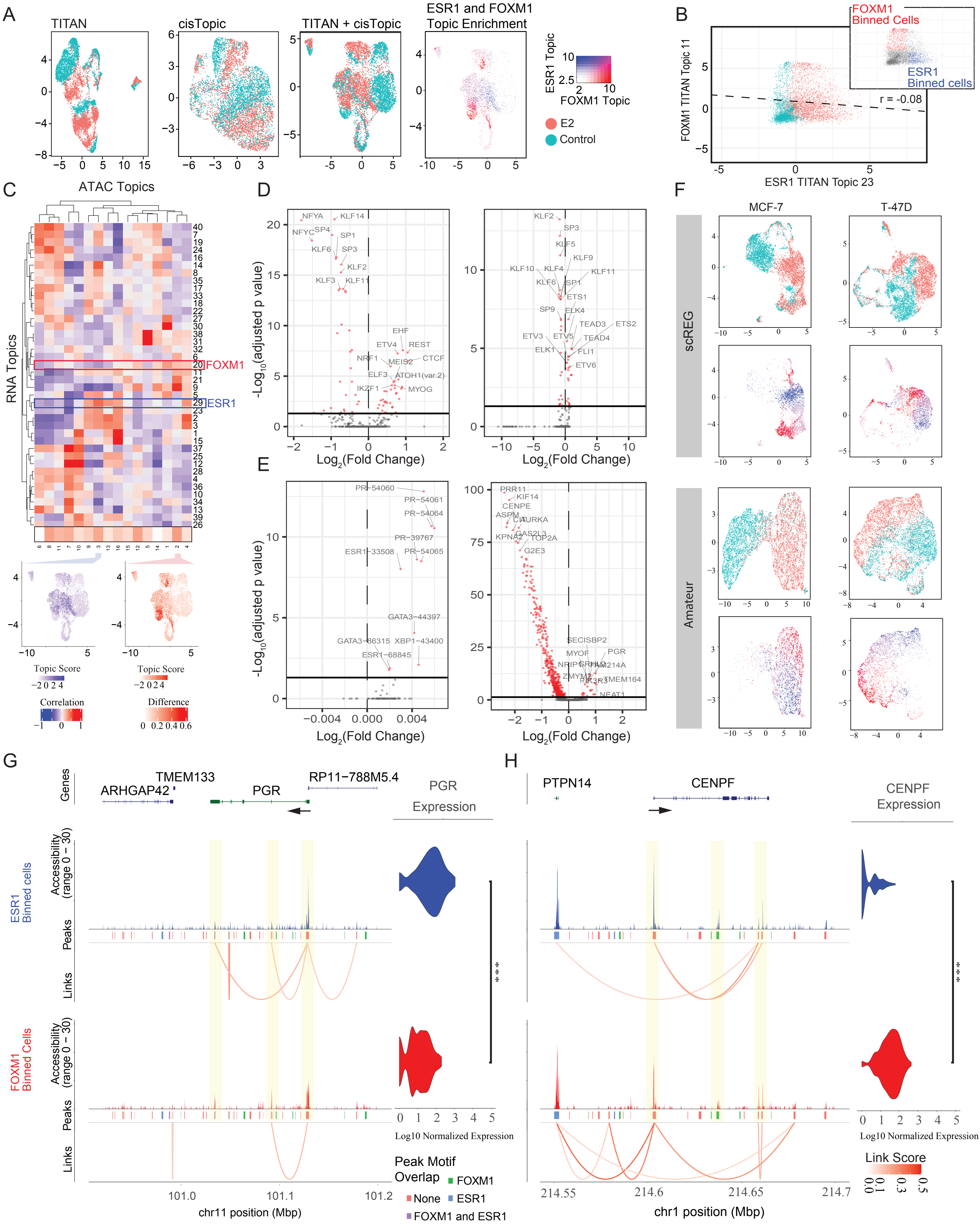

A
TITAN
cisTopic
TITAN + cisTopic
ESR1 and FOXM1 Topic Enrichment
ESR1 Topic
FOXM1 Topic
E2
Control
B
FOXM1 Binned Cells
ESR1 Binned cells
r = -0.08
FOXM1 TITAN Topic 11
ESR1 TITAN Topic 23
C
ATAC Topics
RNA Topics
FOXM1
ESR1
Topic Score
Correlation
Difference
D
-Log10(adjusted p value)
Log2(Fold Change)
E
F
MCF-7
T-47D
scREG
Amateur
G
Genes
ARHGAP42
TMEM133
PGR
RP11−788M5.4
PGR Expression
ESR1 Binned cells
FOXM1 Binned Cells
Accessibility (range 0 − 30)
Peaks
Links
chr11 position (Mbp)
Log10 Normalized Expression
Peak Motif Overlap
FOXM1
None
ESR1
FOXM1 and ESR1
H
PTPN14
CENPF
CENPF Expression
chr1 position (Mbp)
Link Score

## SUPPLEMENTARY FIGURE LEGENDS

**Supplementary Figure S1. (A)** Elbow plots representing rate of perplexity change (RPC) across models of ascending topic numbers in all scRNA-seq datasets used in the study. E2 caption refers to estrogen only treatments and E2/PG caption refers to estrogen and/or progesterone treatments. **(B)** PCA based UMAP (top left) and TITAN based UMAP (bottom left) visualization of the PBMC dataset colored by cell type. Heatmap of TITAN normalized gene topic scores for PBMC classed by cell type (right). **(C)** Overlay of the first 20 PBMC topics on the PCA input UMAP dimensionality reduction plot.

**Supplementary Figure S2. (A)** PCA based UMAPs of estrogen treatments for each cell line and PdXO colored by cluster **(B)** Distribution of cells in PCA based UMAP clusters across estrogen treatment times for each cell line and PdXO. **(C)** 20 TITAN topics in MCF-7 cells overlaid on PCA based UMAP clustering. **(D)** Histogram of TITAN normalized gene topic scores for all topics of MCF-7 E2 only treatment dataset. **(E)** Top enriched pathways for estrogen up- (topic 8) or down- (topic 13) regulated topics in MCF-7 estrogen only treatments in the Bioplanet 2019 database (Enrichr). Significance of overlap p-values is generated by hypergeometric test.

**Supplementary Figure S3. (A)** Spearman correlation between CountClust and TITAN outputs. **(B)** CistromeDB database transcription factor enrichment scores for CountClust ESR1 cluster 14 for MCF-7, 5 for T-47D and 12 for ZR-75-1 estrogen treatment dataset, significance of overlap p-values generated by hypergeometric test**. (C)** Heatmap of top 20 regulons output from SCENIC analysis on E2 treatment datasets. **(D)** Z-score normalized to control comparison for all four tools between corresponding outputs in estrogen treated ZR-75-1 (left) and T-47D (right). **(E)** CPU time and RAM metrics for all three tools compared.

**Supplementary Figure S4. (A)** TITAN based UMAPs of estrogen treatments for each cell line and PdXO colored by cluster **(B)** TITAN heatmaps of estrogen treatments for each cell line and PdXO by treatment.

**Supplementary Figure S5. (A)** Scatter plots representing estrogen up- or downregulated topics. **(B)** Bar plots representing different rates of change in topic enrichment across estrogen treatment lengths.

**Supplementary Figure S6. (A)** PCA based UMAP visualization of scRNA-seq on MCF-7 clonal mix (7,295 cells) vs. clone 1 (5,860 cells) and clone 2 (9,164 cells). UMAP is

colored by clone identity (left) or cluster identity (right). **(B)** Bar plot representation of distribution of clones in each cluster from the PCA based UMAP. **(C)** Heatmap representing TITAN normalized cell topic scores on all clones and treatment conditions. FOXM1 and ESR1 linked topics highlighted. **(D)** Violin plot showing enrichment of topic 2 driven by FOXM1 or topic 8 driven by ESR1 from the MCF-7 cell line treated with estrogen dataset, imputed onto MCF-7 clonal datasets.

**Supplementary Figure S7. (A)** PCA based UMAP visualization of scRNA-seq on MCF-7 (15,315 cells), T-47D (10,668 cells) and ZR-75-1 (9,458 cells) scRNA-seq data after treatments with estrogen and/or progesterone (E2/PG) colored by clusters (top) or treatment (bottom). **(B)** Bar plot representing distribution of cells in PCA based UMAP clusters across treatment timepoints for MCF-7, T-47D and ZR-75-1 cells. **(C)** Heatmap visualization of TITAN topics for MCF-7 and ZR-75-1 cell lines. **(D)** CistromeDB database transcription factor enrichment scores for MCF-7 topic 8 and topic 12 from the estrogen and/or progesterone treatment dataset (E2/PG), significance of overlap p-values generated by hypergeometric test, colored by associated TF.

**Supplementary Figure S8. (A)** Nuclear immunofluorescence staining for DAPI, pFOXM1 and ERα in MCF-7 treated with estrogen for 24h (representative images shown). **(B)** Distribution of ERα protein levels quantified as mean fluorescence intensity within 37,911 nuclei in MCF-7 cells plotted by cell cycle phase and estrogen treatment time imaged by high-content quantitative imaging. Boxplot hinges correspond to the 25th-75th and whiskers correspond to 1.5 * IQR (inter-quartile range) of the hinge. Outlying points are plotted individually. **(C)** Relative gene expression of FOXM1 topic genes in MCF-7 cells with and without 100nM estrogen treatment for 6 and 24 hours.

**Supplementary Figure S9. (A)** PCA based (top) and TITAN based (bottom) UMAP clustering of breast cancer patient sample scRNA-seq dataset by Wu et al. **(B)** Heatmap of TITAN normalized cell topic scores (right) 36,798 epithelial cells colored by subtype and donor. **(C)** Enrichment plots of Imputed topic 11 (FOXM1 driven) and topic 17 (ESR1 driven) in primary tumor BC03 (ER+/Her2+, luminal B, invasive ductal carcinoma) and associated metastatic lymph node BC03LN. **(D)** Enrichment plots of FOXM1 and ESR1 gene expression in primary tumor BC03 and BC03LN. **(E)** Kaplan-Meier survival plots in ER positive breast cancer patients for top 50 genes in topic 17 (left) and topic 11 (right).

**Supplementary Figure S10. Multiomic analysis of gene expression and chromatin accessibility using TITAN and cisTOPIC. (A)** UMAP projection of T-47D (right, 6,615

cells) cell profiles colored by treatment. Projections were calculated using TITAN, cisTopic, and TITAN+cisTopic profiles (see Methods). UMAP projection of TITAN+cisTopic profiles colored by ESR1 and FOXM1 defined by TITAN (far right). Color legend common with panel (B). **(B)** Scatterplot of FOXM1 and ESR1 TITAN topic values in MCF-7. Main figures are colored by treatment with R2 correlation and linear fit shown as a black dashed line. Inset panels show binned FOXM1 and ESR1 topic enriched cells. These bins were defined as cells having ≥20% quantile value for the respective bin and ≤75% quantile value of the opposite bin. This resulted in 904 and 914 T-47D cells for FOXM1 and ESR1 topics, respectively. Color legend shared with panel (A). **(C)** Topic expression heatmap correlation between TITAN and cisTopic topics. TITAN ESR1 (Topic 23, blue rectangle) and FOXM1 (Topic 11, red rectangle) topics are highlighted. Highlighted UMAP projection of TITAN+cisTopic profiles colored by cisTopic topics 3 (left) and 2 (right) showing open chromatin regions specific to TITAN topics (bottom). **(D)** Volcano plot of differential expression (left) and cistrome defined enrichment scores of ChIP-seq peaks (right) between FOXM1 and ESR1 topics for binned T-47D cells defined in panel (D). The top 10 genes or CistromeDB peak sets are shown for FOXM1 and ESR1 bins. Names displayed show genes or target protein and cistrome ID. **(E)** Volcano plot of differential accessibility of transcription factor motifs between FOXM1 and ESR1 for binned MCF-7 (left) and T-47D (right) cells. The top 10 transcription factor motifs for FOXM1 and ESR1 enrichment are shown. For (D) and (E), positive Log2 fold-change reflects ESR1 bin enrichment. Points plotted in red are significant (adjusted p-value ≤ 0.05). **(F)** UMAP projection of MCF-7 (left, 6,438 cells) and T-47D (right, 6,615 cells). In descending order, cell embedding calculated by scREG (see Methods) with cells colored by treatment, and ESR1 and FOXM1 defined TITAN topic values, followed by cell embeddings calculated by Amateur with cells colored by treatment and ESR1 and FOXM1 defined TITAN topic values. Color legend consistent with panel (A). **(G)** Coverage plot of aggregate ATAC profiles for binned T-47D cells defined in panel (B). Genome track covers the gene body of PGR with 5kbp up and downstream. Black arrow represents the transcription direction. Side plot displays the differential gene expression of PGR. Peaks subpanel shows defined open regions on aggregate data, peaks are then colored by overlap with FOXM1 and ESR1 binding motifs. Links subpanel displays cis-coaccessibility linked between peaks, displaying peaks which are correlated. Gray highlight boxes overlap peaks which show at least nominal significance (Logistic Regression, p-value ≤ 0.05). **(H)** Coverage plot as described in panel (G) for genomic location around CENPF. *** denotes adjusted logistic regression p-value ≤ 0.01.

**Supplementary Table 1.** Top 50 genes in TITAN generated topics on all datasets used in this study. MCF7_E2, T47D_E2, ZR751_E2 refer respectively to data from MCF-7, T-47D, ZR-75-1 cell lines treated with estrogen alone. MCF7_E2PG, T47D_E2PG, ZR751_E2PG refer respectively to data from MCF-7, T-47D, ZR-75-1 cell lines treated with estrogen and/or progesterone. MCF7_Cl1_Cl2 refers to the data generated in MCF-7 clones. BC_Chung and BC_Wu correspond to the data generated by Chung et al. and Wu et al. respectively. MCF7_MultiomeRNA and T47D_MultiomeRNA refer to the data generated with the multiome assay in MCF-7 and T-47D cell lines treated with estrogen for 48h.

**Supplementary Table 2.** Top transcription factor enrichment for TITAN generated topics based on CistromeDB analysis in MCF-7, T-47D and ZR-75-1 cell lines treated with estrogen alone (MCF7_E2Top_TF, T47D_E2Top_TF, ZR751_E2Top_TF) or estrogen and or progesterone (MCF7_E2PG_Top_TF, T47D_E2PG_Top_TF, ZR751_E2PG_Top_TF). Id and file names refer to the relevant ChIP studies found in the CistromDB database as explained in methods.

**Supplementary Table 3.** Number of common genes per topic across all topics from scRNA-seq data in MCF-7, T-47D, ZR-75-1, TAMR, HCI003 and HCI003 models treated with estrogen only.

**Supplementary Table 4. ChromVar:** Resulting feature values for logistic regression comparison between ESR1 and FOXM1 binned cells chromVAR values for both MCF-7 and T-47D cell lines. **Cistrome:** Resulting feature values for logistic regression comparison between ESR1 and FOXM1 binned cells cistrome enrichment values for both MCF-7 and T-47D cell lines. **GeneExp:** Resulting feature values for logistic regression comparison between ESR1 and FOXM1 binned cells SCT-normalized RNA count values for both MCF-7 and T-47D cell lines. cell_line: Cell line [MCF-7, T-47D] used in comparison. chromvar_motif_label: Feature name for chromVAR comparison. Names are in JASPAR2020 format. p_val: P value calculated through logistic regression using default flags with FindMarkers function in Seurat. avg_log2FC: Average Log2 Fold Change calculated through logistic regression using default flags with FindMarkers function in Seurat. pct.1: Group 1 percentage value used in logistic regression. Group 1 is ESR1 binned cells. pct.2 : Group 2 percentage value used in logistic regression. Group 2 is FOXM1 binned cells. p_val_adj: Adjusted p-value calculated using Bonferroni correction. chromvar_gene_name: Gene name associated with JASPAR2020 formated name in column "chromvar_motif_label", converted to gene name for readability. Sig:

Binary value [sig, nonsig] used to color points in volcano plots. "sig" denotes a p_val_adj value of less than or equal to 0.05. label: Label is used for identifying features plotted on the resultant volcano plot. Top 10 features (defined by p_val_adj) for enrichment in either bin is used. cistrome_name: Cistrome name in the format [Cell Line of Origin]-[Target Protein for ChIPseq]-[cistromeID]. gene_name: Gene name assigned to RNA values.
